# LYRA, a webserver for lymphocyte receptor structural modeling

**DOI:** 10.1093/nar/gkv535

**Published:** 2015-05-24

**Authors:** Michael Schantz Klausen, Mads Valdemar Anderson, Martin Closter Jespersen, Morten Nielsen, Paolo Marcatili

**Affiliations:** 1 Center for Biological Sequence Analysis, Technical University of Denmark, Kgs. Lyngby, Denmark; 2 Instituto de Investigaciones Biotecnológicas, Universidad Nacional de San Martín, Buenos Aires, Argentina

## Abstract

The accurate structural modeling of B- and T-cell receptors is fundamental to gain a detailed insight in the mechanisms underlying immunity and in developing new drugs and therapies. The LYRA (LYmphocyte Receptor Automated modeling) web server (http://www.cbs.dtu.dk/services/LYRA/) implements a complete and automated method for building of B- and T-cell receptor structural models starting from their amino acid sequence alone. The webserver is freely available and easy to use for non-specialists. Upon submission, LYRA automatically generates alignments using *ad hoc* profiles, predicts the structural class of each hypervariable loop, selects the best templates in an automatic fashion, and provides within minutes a complete 3D model that can be downloaded or inspected online. Experienced users can manually select or exclude template structures according to case specific information. LYRA is based on the canonical structure method, that in the last 30 years has been successfully used to generate antibody models of high accuracy, and in our benchmarks this approach proves to achieve similarly good results on TCR modeling, with a benchmarked average RMSD accuracy of 1.29 and 1.48 Å for B- and T-cell receptors, respectively. To the best of our knowledge, LYRA is the first automated server for the prediction of TCR structure.

## INTRODUCTION

The immune system has the ability to target and fight with extreme efficacy and specificity dangerous molecules of either exogenous (pathogens, toxins) or self-origin (tumors, metabolic by-products). Two key molecules in this extraordinary defence mechanism are T-cell (TCRs) and B-cell receptors (BCRs, antibody or immunoglobulins) that with their interplay ensure a precise yet controlled immune response. In order to do so, the lymphocyte cells specific for their production have the capacity, unique among all the cell types in higher organisms, to control their genomic sequence by means of genomic recombination, to be subjected to positive or negative selection according to their ability to recognize self and non-self molecules, and in the case of B-cells, to further change their genomic content in a process called ‘affinity maturation’. All these events eventually allow the organism to generate a huge yet highly controlled repertoire of different lymphocyte receptors (1).

Antibodies recognize potentially harmful molecules (antigens) present in the blood or in mucosal tissue and represent one of the first barriers against infection. The ability to study and predict their structure has been of fundamental importance to our understanding of the immune system, of pathogenic and autoimmune diseases (2–4) and for the development of new therapies and drugs (5). Even though the diversity of the antibodies produced in a single person is larger than that of all other human proteins altogether (6), we can predict their structure with extreme accuracy (7,8). This exceptional ability derives from a fundamental discovery that Chothia and Lesk made 30 years ago (9–11): although T- and B-cell receptors have a very large sequence variability, especially in their antigen-binding site (ABS), this does not have a comparable effect on their main chain conformation that is very conserved. The six CDR (complementarity determining regions) loops that compose the ABS (antibody binding site) can only assume a limited amount of conformations named *canonical structures* that can usually be identified by specific sequence features. The canonical structure model has been proven valid for both T- and B-cell receptors, but it has until now been developed and implemented into automated modeling tools only for B cell receptor (BCR) molecules. The reasons for this bias reside in the larger number of antibodies with an available solved structure and in the large use of antibodies in pharmaceutical and industrial applications. In the last few years however, the T cell receptors (TCR) have gained increased attention for the development of vaccines and therapies to treat cancers (12,13), allergies and autoimmune disorders (14,15), and the precise modeling of their structure has become a fundamental step for the advancement of the field.

Here, we present LYRA (LYmphocyte Receptor Automated modeling), a web server for automated modeling of both B- and T-cell receptors. It is based on the canonical structure method and can produce, easily and within minutes, extremely reliable models of lymphocyte receptors.

## MATERIALS AND METHODS

### Modeling pipeline

The input sequences are scanned with sequence profiles generated in-house. For each input sequence, the best-scoring profile is used to infer the receptor and chain type and the correct alignment. If LYRA can identify and properly align both chains of a given lymphocyte receptor, the pipeline continues with an automatic selection, from a database of curated templates, of the best framework template and eventually of the CDR templates that need to be grafted. The templates are then merged and the side chains are repacked to generate the final model. The overall modeling procedure takes on average less than a minute (5 s queuing time, 30 s computing time). A more detailed description of each modeling step follows below.

### Template database

PDB codes of all BCR and TCR structures present in the IMGT/3Dstructure-DB (16) were retrieved and culled using the Pisces web server (17) to remove all redundant structures. Any complete molecule with at least one non-redundant chain was retained in the database, as well as non-redundant non-paired chains. The resulting TCR structure database consists of 105 paired chains, two individual α chain and nine individual β chain structures. The BCR structure database consists of 846 complexed chain pairs, 10 individual heavy chains, five individual kappa chains and one individual lambda chain. All the structures have been aligned and renumbered using ad hoc sequence profiles (see next paragraph). The template database is automatically updated on a monthly basis. All novel solved structures deposited in the PDB database (http://www.rcsb.org/pdb/) are checked and the ones that are non-redundant with any molecule in the current version of the database are added to it (see Supplementary Materials for details).

### Sequence profiles

LYRA uses sequence profiles for BCR heavy, lambda and kappa chains and TCR α and β chains to identify and align the lymphocyte receptor sequences. The BCR HMMs were generated according to a protocol previously developed by the authors (18). The final HMMs contain 5462, 12 930 and 36 895 sequences for the lambda, kappa and heavy chain respectively, aligned according to the Kabat-Chothia numbering method with the additional insertions described by Abhinandan and Martin (19).

To generate HMM profiles for TCR α and β chains, we produced a multiple structural alignment of the 212 α chain and 221 β chain structures retrieved in the previous step (excluding redundancy reduction) using the 3DCoffee/TMalign mode of the t-coffee software version 11.0010 (20). The multiple sequence alignments (MSAs) extracted from these structural alignments were then used as input for HMMER version 3.1 (21) (hmmbuild, default settings) to create seed HMM profiles. Additional nucleic acid sequences for α and β chains were then downloaded from the IMGT/LIGM-DB (22), translated to amino acid sequences and aligned using the seed HMM profiles. All the hits longer than 100 residues, with an E-value below 10^−50^ (501 α chain and 599 β chain sequences) were added to the corresponding MSA. Consistently with what we observe in the Kabat-Chothia alignment (10), the insertions in the CDR regions of the MSAs were then right-aligned in order to have a single re-entry position in the C-terminal region of each CDR alignment. Insertions in the framework regions were not edited. The resulting alignments were used to generate the final HMMs.

### Canonical structures

Canonical structures (CSs) and corresponding rules for BCR models were previously defined by others and us (10,11,23–28). To generate CS classes and prediction rules for TCR CDR loops, we adapted the method from North et al. (29). CDR loops were grouped by length, and the distance between each pair of loops of the same length was calculated as the sum of the distances of the phi and psi angles of each residue in the two loops, the distance being defined as *D*(θ_1_, θ_2_) = 2(1 − cos(θ_1_ − θ_2_)). The CSs were obtained by applying the affinity propagation clustering algorithm from the Python package scikit-learn (30) to the distance matrices obtained for each chain type, CDR and loop length. The resulting CSs for TCR α and β chains are reported in Supplementary Tables S1 and S2 respectively. Following Chailyan *et al*. (23,24), we trained a sequence-based prediction method that, given the sequence of a TCR chain, can predict the CS of its CDRs. More details about the TCR canonical structures (Supplementary Tables S1 and S2), the procedure for CS predictions and its accuracy (Supplementary Tables S3–S5 and Supplementary Figures S1–S3) can be found in the Supplementary Materials.

### Template selection

As previously shown, the template selection step is crucial for the model accuracy, depending on the compromise between higher sequence similarity on the one hand and the need to merge together regions from different templates on the other hand (18). To this aim, we use a combination of different scores and selection procedures. Given an aligned target sequence, we calculate five scores for each template. The first four scores are similarity scores, one for the complete sequence and one for each of the three CDRs, using the BLOSUM62 similarity matrix. Finally, there is a *combined score*, calculated as the sum of the complete sequence score with the scores of the template CDRs that match the corresponding target CDR canonical structure.

In order to favor framework templates from the same crystal structure, thus avoiding errors or clashes that might be introduced by packing together different chains, the following pipeline is used: for each target chain, the 20 templates with the highest combined score and a sequence identity with the target of at least 60% are listed and the templates coming from the same crystal structure and with the highest overall sequence identity is selected. If no pair of templates from the same crystal structure can be found, the program falls back to select for each individual chain the framework template with the highest combined loop score, thus minimizing the number of CDR loops that have eventually to be grafted in the model.

Next, each loop (with the exception of β CDR3) in each query chain is scanned. If the canonical structure of the loop in the selected framework template matches the calculated canonical structure for the query sequence, the loop is left untouched, otherwise a loop with the same canonical structure and the highest CDR-specific score is selected as template. In the cases (template blacklisting, no canonical structure predicted) where no such loop can be found in the database the template loop with the same length of the template loop and the highest CDR score is selected. This selection method is always used for the β CDR3 loops, for which no clear CS could be identified.

### Model assembly

The final model assembly consists of three stages: packing of the two chains, loop grafting and side chain modeling. If the selected framework templates for both chains originate from different crystal structures, a set of interface packing residues is used to construct a ‘pseudo-sequence’ based on positions that determine the interface packing. The pair of templates from the same crystal structure and with the highest BLOSUM62 similarity based on the pseudo-sequence is chosen and the two template chains are modeled superimposing the target and template residues of all residues belonging to the interface (18,23). Template loops from non-framework templates are grafted by superimposing the backbone atoms of the two residues before the N-terminal residue of the loop and the two residues immediately after the C-terminal of the loop. Then, the sidechains of CDR3 residues and of each residue not conserved between target and template are repacked using the Scwrl4 software (31). Finally, in order to remove clashes and bad geometries, the model is subjected to 500 steps of energy minimization using the ENCAD program (32).

## WEB INTERFACE

The LYRA (http://www.cbs.dtu.dk/services/LYRA/) provides an easy-to-use interface to input the amino acid sequence data, a result page containing graphical and downloadable output of the model and an advanced modeling options for experienced users, and a detailed help page including sample sequences and output. In the following, we will describe the main elements of the LYRA web server in more detail. Further information and a more detailed guide can be found on the LYRA help page.

### Input page

The main input of the LYRA input page consists in the amino acid sequence of both chains of a TCR or a BCR. Each sequence should be copied in one of the two text areas, labeled ‘Chain 1 Protein Sequence’ and ‘Chain 2 Protein Sequence’ in raw format (i.e. with no header or special character). The ‘PDB Blacklist’ input area (optional) allows the user to enter a comma-separated list of any PDB structure that should not be used as template and therefore discarded in the modeling pipeline. The two buttons labeled ‘TCR’ and ‘BCR’ will load the text areas with sample BCR or TCR sequences respectively, overwriting any sequence already copied in the input areas. Upon pressing the ‘submit’ the user will be redirected to a queue page that will reload until job completion. The user can optionally submit his/her email address in this page and receive a mail with a link to the job. This field is not compulsory and it is sufficient to bookmark the page to access the results at a later time.

### Output page

The LYRA output page contains a header bar linking to several tabs. The ‘Summary’ tab shows detailed information on the modeling process: the templates used to model the framework and the CDR loops of each chain are reported for both chains. For each CDR loop, the CS of the target and of the corresponding template is shown. A complete list of the CS can be displayed by clicking on the button with a question mark button near the corresponding chain type. The user can visualize the model either interactively via the JSMol application in the ‘View Structure’ tab or with the high-resolution renderings generated using the PyMOL software in the ‘Structure Images’ tab. In both cases, the CDR loops are highlighted using different colors. The PDB file with the complete energy-minimized model can be inspected online in the ‘PDB File’ tab or downloaded by clicking on the ‘Download PDB File’ button. It is worth noticing that the header section of the pdb file generated by LYRA contains most of the modeling details found on the output page. The ‘Log’ tab displays technical information on the job inputs, outputs and possible errors.

In the ‘Manual Template Selection’ the user can override the automatic selection of framework templates by clicking on any of the templates listed in the page. For each template all the relevant information are listed, such as the canonical structure of their CDR loops, the sequence identity and Blosum score with respect to the target sequence. An alignment of the target sequences with the templates currently selected is visible in the bottom part of the page. By clicking the ‘Submit’ button in this tab a new job will be launched, using the framework templates just selected and the automatically selected templates for the CDR loops. Additional files can be downloaded by clicking on the rightmost button in the header bar labeled ‘More’. These files include a complete summary of the input molecules and of the modeling parameters in JSON and csv format, a PyMOL script containing the model colored similarly to the pictures displayed in the ‘Structure Images’ tab, a ‘Pre-SCWRL’ pdb in which only backbone atoms and side-chains conserved from the templates are present and a non-minimized complete model, to be used as input for sidechain modeling and energy minimization software of the user's choice.

### Help page

The LYRA server provides a comprehensive help page that can be accessed from the header bars in the input and output pages. The help page contains a short description of the LYRA server and its usage, an outline of the modeling pipeline, a detailed definition of framework and CDR regions in each lymphocyte receptor chain, links to the tables with the CS definition in each chain type, and a description of the server outputs.

## EVALUATION AND CASE STUDY

In order to test the accuracy of LYRA, we applied a leave-one-out procedure to all the paired TCR and BCR molecules in our template database. In each round, we removed from the database the molecule being modeled together with any template having a sequence similarity of 90% or higher with the molecule itself. The overall results of the assessment are shown in Figure 2, panels A and B. The accuracy of the server was found to be high for both TCR and BCR modeling (TCRs: 1.48 Å global RMSD, 2.13 Å binding site RMSD, and BCR: 1.29 Å global RMSD, 2.24 Å binding site RMSD). The values for BCR modeling are at par with values obtained using state-of-the-art tools for antibody structure prediction (33). More details on the accuracy of the method are reported in the supplementary materials (Supplementary Figure S4 and Supplementary Tables S6 and S7).

**Figure 1.**
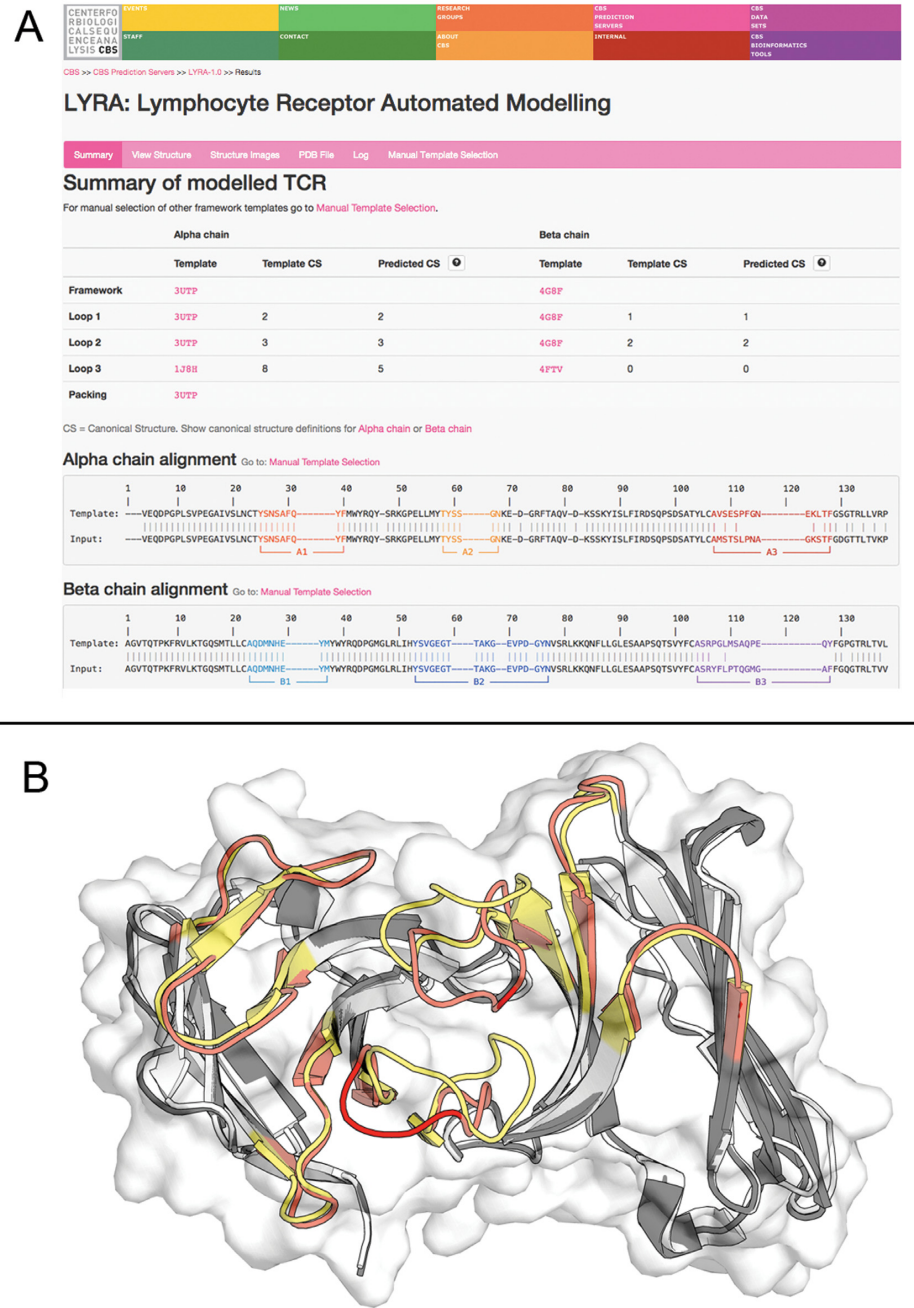
Modeling results for the 4x6B example. (**A**) The Summary output page contains the target predicted CSs and the final target-template alignment with CDR regions highlighted in different colors. (**B**) The corresponding model (dark grey framework, red CDRs) superposed to the 4x6B solved structure (white framework, yellow CDRs).

**Figure 2.**
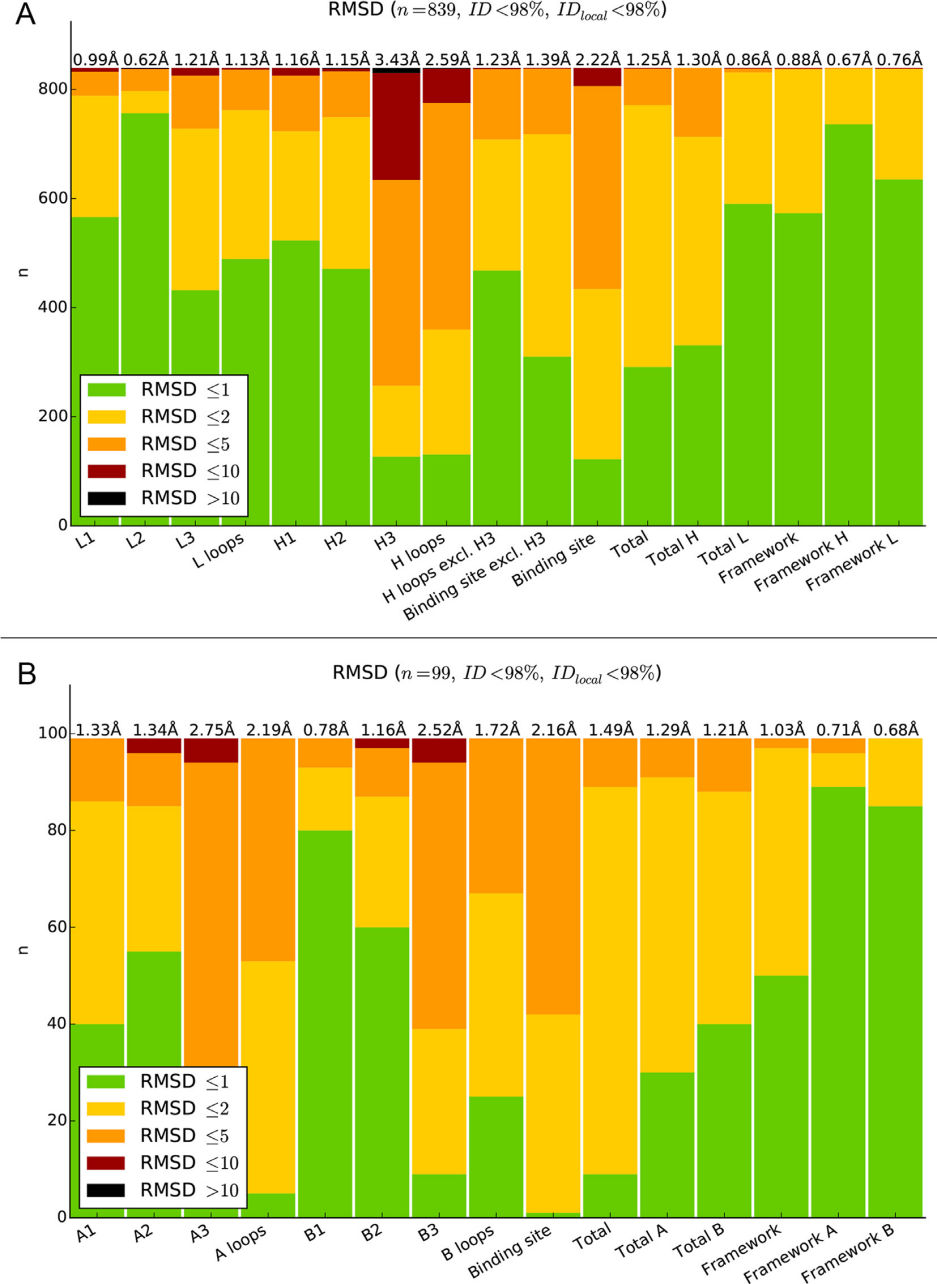
Leave-one-out assessment of LYRA models for BCR (**A**) and TCR molecules (**B**). Each bar plot represents the number of models for which a given region (reported on the x-axis) had a C_α_ RMSD to the corresponding region of the solved structure below 1 Å (green), between 1 and 2 Å (yellow), between 2 and 5 Å (orange), between 5 and 10 Å (red) or exceeding 10 Å (black). The overall average RMSD per region is reported on top.

### Comparison with similar methods

While several other antibody automated modeling tools are present (18,34–39), LYRA is to the best of our knowledge the first publicly available method dedicated to TCR modeling. A recent published paper assessed the accuracy of the majority of currently available antibody modeling software and servers, both commercial and academic (33). We used LYRA to model all the antibodies used in this assessment with the same redundancy reduction used in the leave-one-out procedure, and the results are shown in Table 1. We can observe that LYRA builds in a completely automated fashion models with accuracy comparable to manually curated and optimized models. Only for the rabbit antibody Ab01 (PDB code: 4MA3) does LYRA fail to produce a model due to the unusual insertions in the FR3 region of its heavy chain.

**Table 1.** Evaluation of LYRA antibody models on the AMA-II dataset

ALIGNED	All	FR	FRH	FRL	FRH
RMSD	All	CDR	CDRH	CDRL	H3
Ab02 (4KUZ)	1.40 Å (1.3 Å)	2.53 Å (2.0 Å)	3.66 Å (2.4 Å)	0.81 Å (0.8 Å)	5.35 Å (3.4 Å)
Ab03 (4KQ3)	1.32 Å (1.0 Å)	1.95 Å (1.5 Å)	2.51 Å (1.9 Å)	0.83 Å (0.5 Å)	2.70 Å (2.2 Å)
Ab04 (4KQ4)	1.75 Å (1.1 Å)	3.40 Å (1.2 Å)	4.23 Å (1.4 Å)	0.71 Å (0.5 Å)	1.78 Å (1.8 Å)
Ab05 (4M6M)	1.33 Å (1.3 Å)	1.91 Å (1.7 Å)	2.00 Å (2.0 Å)	1.53 Å (2.3 Å)	3.31 Å (2.8 Å)
Ab06 (4M6O)	1.27 Å (1.2 Å)	2.59 Å (1.8 Å)	3.28 Å (1.7 Å)	0.55 Å (0.6 Å)	4.48 Å (2.3 Å)
Ab07 (4MAU)	1.64 Å (0.6 Å)	3.00 Å (1.1 Å)	3.45 Å (0.8 Å)	1.81 Å (1.4 Å)	5.30 Å (1.0 Å)
Ab08 (4M7K)	1.55 Å (0.9 Å)	3.27 Å (1.6 Å)	4.09 Å (1.8 Å)	0.55 Å (1.1 Å)	6.02 Å (2.4 Å)
Ab09 (4KMT)	0.62 Å (0.7 Å)	0.98 Å (1.2 Å)	1.00 Å (1.5 Å)	0.49 Å (0.6 Å)	1.35 Å (2.0 Å)
Ab10 (4M61)	1.12 Å (0.9 Å)	2.04 Å (1.5 Å)	2.21 Å (1.8 Å)	1.04 Å (1.1 Å)	3.20 Å (2.5 Å)
Ab11 (4M43)	1.26 Å (1.0 Å)	1.24 Å (1.4 Å)	1.56 Å (1.6 Å)	0.63 Å (0.5 Å)	2.32 Å (2.2 Å)

The RMSD between the LYRA model and the corresponding solved structure (reported between brackets in the first column) is calculated on the backbone atoms of the region reported in the RMSD row after superimposing the backbone atoms of the region reported in the ALIGNED row. The corresponding RMSD for the JOA group (32), that was amongst the best competitors in the AMA-II assessment (7), is reported in brackets.

### Case study

To demonstrate the functionality of the LYRA web server also for TCR modeling, we show the modeling of 4×6B (40), a TCR molecule recently published in the PDB database and with a sequence similarity with the closest templates in our database of 86.5% for the α and 90.4% for the β chain with any TCR molecule. The model generated by LYRA, superposed to the 4x6B crystal structure, is shown in Figure 1 panel B. It has a global RMSD with the target structure of 1.61 Å, and the binding site RMSD after framework superposition is 2.75 Å. As apparent from the figure, the model has an overall good quality, with most of the inaccuracy being concentrated in the CDR3 regions of both chains. An overview of the method accuracy on an independent set of TCR molecules not used at any point in this work can be found in Supplementary Table S8.

## CONCLUSIONS

We present the LYRA webserver, a completely automated method for the prediction of BCR and TCR structures. LYRA is, to the best of our knowledge, the first automated modeling tool for TCRs. Given its extremely simple interface, we believe LYRA will help researchers with limited knowledge of bioinformatics and computational tools, to easily build reliable models of lymphocyte receptors. The quality of the models generated by LYRA is at par with other methods and sufficient to make them useful in a vast number of biotechnological, pharmaceutical and computational tasks. As expected (25,26,36), the weak point of the pipeline is the modeling of the third CDR loop of heavy, α and β chain, either because a proper template loop is not available or because the adopted sequence-based template selection scheme fails to select it. While some improvements can be achieved in the short term (e.g. by including the predicted interactions between neighbouring CDRs in the process of template selection), we believe that these issues will become less dramatic in future, since more and more BCR and TCR solved structures are becoming available thus increasing the number of available templates and allowing to develop more sophisticated template selection methods.

## SUPPLEMENTARY DATA

Supplementary Data are available at NAR Online.

SUPPLEMENTARY DATA

## References

[1] Janeway C. (2005). Immunobiology : The Immune System in Health and Disease.

[2] Marcatili P., Ghiotto F., Tenca C., Chailyan A., Mazzarello A.N., Yan X.J., Colombo M., Albesiano E., Bagnara D., Cutrona G. (2013). Igs expressed by chronic lymphocytic leukemia B cells show limited binding-site structure variability. J. Immunol..

[3] Ghiotto F., Marcatili P., Tenca C., Calevo M.G., Yan X.J., Albesiano E., Bagnara D., Colombo M., Cutrona G., Chu C.C. (2011). Mutation pattern of paired immunoglobulin heavy and light variable domains in chronic lymphocytic leukemia B cells. Mol. Med..

[4] Zibellini S., Capello D., Forconi F., Marcatili P., Rossi D., Rattotti S., Franceschetti S., Sozzi E., Cencini E., Marasca R. (2010). Stereotyped patterns of B-cell receptor in splenic marginal zone lymphoma. Haematologica.

[5] Shirai H., Prades C., Vita R., Marcatili P., Popovic B., Xu J., Overington J.P., Hirayama K., Soga S., Tsunoyama K. (2014). Antibody informatics for drug discovery. Biochim. Biophys. Acta.

[6] Glanville J., Zhai W., Berka J., Telman D., Huerta G., Mehta G.R., Ni I., Mei L., Sundar P.D., Day G.M. (2009). Precise determination of the diversity of a combinatorial antibody library gives insight into the human immunoglobulin repertoire. Proc. Natl. Acad. Sci. U.S.A..

[7] Almagro J.C., Teplyakov A., Luo J., Sweet R.W., Kodangattil S., Hernandez-Guzman F., Gilliland G.L. (2014). Second antibody modeling assessment (AMA-II). Proteins.

[8] Almagro J.C., Beavers M.P., Hernandez-Guzman F., Maier J., Shaulsky J., Butenhof K., Labute P., Thorsteinson N., Kelly K., Teplyakov A. (2011). Antibody modeling assessment. Proteins.

[9] Al-Lazikani B., Lesk A.M., Chothia C. (2000). Canonical structures for the hypervariable regions of T cell alphabeta receptors. J. Mol. Biol..

[10] Al-Lazikani B., Lesk A.M., Chothia C. (1997). Standard conformations for the canonical structures of immunoglobulins. J. Mol. Biol..

[11] Chothia C., Lesk A.M. (1987). Canonical structures for the hypervariable regions of immunoglobulins. J. Mol. Biol..

[12] Kershaw M.H., Westwood J.A., Slaney C.Y., Darcy P.K. (2014). Clinical application of genetically modified T cells in cancer therapy. Clin. Transl. Immunol..

[13] Kershaw M.H., Westwood J.A., Darcy P.K. (2013). Gene-engineered T cells for cancer therapy. Nat. Rev. Cancer.

[14] Curotto de Lafaille M.A., Lafaille J.J. (2002). CD4(+) regulatory T cells in autoimmunity and allergy. Curr. Opin. Immunol..

[15] Vandenbark A.A., Morgan E., Bartholomew R., Bourdette D., Whitham R., Carlo D., Gold D., Hashim G., Offner H. (2001). TCR peptide therapy in human autoimmune diseases. Neurochem. Res..

[16] Ehrenmann F., Kaas Q., Lefranc M.P. (2010). IMGT/3Dstructure-DB and IMGT/DomainGapAlign: a database and a tool for immunoglobulins or antibodies, T cell receptors, MHC, IgSF and MhcSF. Nucleic Acids Res..

[17] Wang G., Dunbrack R.L. Jr (2005). PISCES: recent improvements to a PDB sequence culling server. Nucleic acids Res..

[18] Marcatili P., Olimpieri P.P., Chailyan A., Tramontano A. (2014). Antibody structural modeling with prediction of immunoglobulin structure (PIGS). Nat. Protoc..

[19] Abhinandan K.R., Martin A.C. (2008). Analysis and improvements to Kabat and structurally correct numbering of antibody variable domains. Mol. Immunol..

[20] O'Sullivan O., Suhre K., Abergel C., Higgins D.G., Notredame C. (2004). 3DCoffee: combining protein sequences and structures within multiple sequence alignments. J. Mol. Biol..

[21] Finn R.D., Clements J., Eddy S.R. (2011). HMMER web server: interactive sequence similarity searching. Nucleic Acids Res..

[22] Giudicelli V., Duroux P., Ginestoux C., Folch G., Jabado-Michaloud J., Chaume D., Lefranc M.P. (2006). IMGT/LIGM-DB, the IMGT comprehensive database of immunoglobulin and T cell receptor nucleotide sequences. Nucleic Acids Res..

[23] Chailyan A., Marcatili P., Tramontano A. (2011). The association of heavy and light chain variable domains in antibodies: implications for antigen specificity. FEBS J..

[24] Chailyan A., Marcatili P., Cirillo D., Tramontano A. (2011). Structural repertoire of immunoglobulin lambda light chains. Proteins.

[25] Kuroda D., Shirai H., Kobori M., Nakamura H. (2009). Systematic classification of CDR-L3 in antibodies: implications of the light chain subtypes and the VL-VH interface. Proteins.

[26] Kuroda D., Shirai H., Kobori M., Nakamura H. (2008). Structural classification of CDR-H3 revisited: a lesson in antibody modeling. Proteins.

[27] Shirai H., Kidera A., Nakamura H. (1996). Structural classification of CDR-H3 in antibodies. FEBS Lett..

[28] Chothia C., Lesk A.M., Tramontano A., Levitt M., Smith-Gill S.J., Air G., Sheriff S., Padlan E.A., Davies D., Tulip W.R. (1989). Conformations of immunoglobulin hypervariable regions. Nature.

[29] North B., Lehmann A., Dunbrack R.L. Jr (2011). A new clustering of antibody CDR loop conformations. J. Mol. Biol..

[30] Pedregosa F., Varoquaux G., Gramfort A., Michel V., Thirion B., Grisel O., Blondel M., Prettenhofer P., Weiss R., Dubourg V. (2011). Scikit-learn: machine learning in python. J. Mach. Learn. Research.

[31] Krivov G.G., Shapovalov M.V., Dunbrack R.L. Jr (2009). Improved prediction of protein side-chain conformations with SCWRL4. Proteins.

[32] Levitt M., Hirshberg M., Sharon R., Daggett V. (1995). Potential energy function and parameters for simulations of the molecular dynamics of proteins and nucleic acids in solution. Comput. Phys. Commun..

[33] Teplyakov A., Luo J., Obmolova G., Malia T.J., Sweet R., Stanfield R.L., Kodangattil S., Almagro J.C., Gilliland G.L. (2014). Antibody modeling assessment. II. Structures and models. Proteins.

[34] Yamashita K., Ikeda K., Amada K., Liang S., Tsuchiya Y., Nakamura H., Shirai H., Standley D.M. (2014). Kotai Antibody Builder: automated high-resolution structural modeling of antibodies. Bioinformatics.

[35] Weitzner B.D., Kuroda D., Marze N., Xu J., Gray J.J. (2014). Blind prediction performance of RosettaAntibody 3.0: grafting, relaxation, kinematic loop modeling, and full CDR optimization. Proteins.

[36] Messih M.A., Lepore R., Marcatili P., Tramontano A. (2014). Improving the accuracy of the structure prediction of the third hypervariable loop of the heavy chains of antibodies. Bioinformatics.

[37] Sircar A., Kim E.T., Gray J.J. (2009). RosettaAntibody: antibody variable region homology modeling server. Nucleic Acids Res..

[38] Marcatili P., Rosi A., Tramontano A. (2008). PIGS: automatic prediction of antibody structures. Bioinformatics.

[39] Whitelegg N.R., Rees A.R. (2000). WAM: an improved algorithm for modelling antibodies on the WEB. Protein Eng..

[40] Birkinshaw R.W., Pellicci D.G., Cheng T.Y., Keller A.N., Sandoval-Romero M., Gras S., de Jong A., Uldrich A.P., Moody D.B., Godfrey D.I. (2015). alphabeta T cell antigen receptor recognition of CD1a presenting self lipid ligands. Nat. Immunol..

