# Supplementary material for: LYRA, a webserver for lymphocyte receptor structural modeling
Source: Nucleic Acids Res. 2015 May 24;43(Web Server issue):W349–55. doi: 10.1093/nar/gkv535 (PMC4489227; doi:10.1093/nar/gkv535)
Supplement: SUPPLEMENTARY DATA [file supp_43_W1_W349__index.html]

LYRA, a webserver for lymphocyte receptor structural modeling — LYRA, a webserver for lymphocyte receptor structural modeling — SUPPLEMENTARY DATA 

# LYRA, a webserver for lymphocyte receptor structural modeling

## SUPPLEMENTARY DATA

- SUPPLEMENTARY DATA
