# Supplementary material for: LYRA, a webserver for lymphocyte receptor structural modeling
Source: Nucleic Acids Res. 2015 May 24;43(Web Server issue):W349–55. doi: 10.1093/nar/gkv535 (PMC4489227; doi:10.1093/nar/gkv535)
Supplement: SUPPLEMENTARY DATA [file supp_gkv535_nar-00585-web-b-2015-File004.docx]

**Supplementary Materials**

**LYRA, a webserver for lymphocyte receptor structural modeling**

**Michael Schantz Klausen^1^, Mads Valdemar Anderson^1^**, **Martin Closter Jespersen^1^, Morten Nielsen^1,2^, Paolo Marcatili^1^**

[sch@ntz.nu](mailto:sch@ntz.nu), [mvand@bio.dtu.dk](mailto:mvand@bio.dtu.dk), [martincj@cbs.dtu.dk](mailto:martincj@cbs.dtu.dk), [mniel@cbs.dtu.dk](mailto:mniel@cbs.dtu.dk),[pmar@cbs.dtu.dk](mailto:pmar@cbs.dtu.dk)

**1:** Center for Biological Sequence Analysis, Technical University of Denmark, Kgs. Lyngby, Denmark

**2:** Instituto de Investigaciones Biotecnológicas, Universidad Nacional de San Martín, Buenos Aires, Argentina

1. **TCR Canonical Structures**

Canonical structures (CS) and corresponding rules for BCR models were previously defined by others and us (1-8). To generate CS classes and prediction rules for TCR CDR loops, we adapted the method from North et al. (9). CDR loops were grouped by length, and the distance between each pair of loops of the same length was calculated as the sum of the distances of the phi and psi angles of each residue in the two loops, the distance being defined as D(θ_1_, θ_2_) = 2(1 − cos(θ_1_ − θ_2_)). The CS’s were obtained by applying the affinity propagation clustering algorithm from the Python package scikit-learn (10) to the distance matrices obtained for each chain type, CDR and loop length. The resulting CS for TCR α and β chains are reported in supplementary table 1 and 2 respectively. Following Chailyan et al. (1,2), we developed a sequence-based method to predict the CS of a given TCR given its sequence alone. For each chain type, CDR and CDR length, we used the HMM based alignment of the TCR sequences together with the CS derived from the structure based clustering to train a Random Forest from the Python package scikit-learn (10). The accuracy of such predictors (defined as the fraction of times the correct CS is predicted from the sample sequence alone), calculated using the out-of-bag cross-validation of the random forest method, is of 97.20% (A1), 85.98% (A2), 63.21% (A3), 98.25%(B1), 95.58%(B2) and 46.43% (B3).

Given the very poor results of the CS method for the B3 loop we do not use the B3 CSs in selecting the templates but a simple Blosum62 sequence similarity score on B3 templates with a similar length.

Finally, we tested the effect on the model accuracy of using CSs rather than a simple sequence-similarity search (calculated using the Blosum62 similarity matrix on the HMM based alignment of the CDR sequences) for CDR template selection. The results (Supplementary figure S1) show that there is a small but consistent improvement in the model accuracy when the CS selection method is used.

Given the limited amount of TCR structure, we assessed how many functional TCR variable genes can be modeled by the CS we defined. To this aim, we downloaded the sequences of all TCR α and β functional variable gene present in the IMGT-GENE-DB database, we calculated their predicted CS and the number of templates in our structural database with a similar CDR length (Same length columns) and with the same CS (Same CS column). The results, shown in tables S3, S4 and S5, show that approximately 13% the IMGT TRAV, and 5% of the IMGT TRBV germline sequences lack a proper template for either their CDR1 or CDR2 region and therefore can not be properly modeled using the current version of the method.

1. **Evaluation**

We evaluated the accuracy of the LYRA webserver using two different strategies: a leave-one-out validation on the template structural database with a similarity threshold of 95% and 90% identity on the selected templates and an evaluation on an independent dataset not used at any point in the current work.

The leave-one-out validation has been performed by removing, one at the time, each structure from the template dataset together with any other template with a sequence identity grater than 95% or 90% according to the identity threshold. The structure removed has then been modeled using the remaining template and finally the raw and refined models have been compared to it. We measured the global and framework RMSD on all the backbone atoms. The RMSD for the loops has been calculated on the backbone of the CDR region of interest after superposing the corresponding framework region. The ABS RMSD has been calculated on the backbone atoms of all the CDRs after superposing the framework of both chains.

The results for TCR molecules using the 95% and 90% thresholds are reported in table S6 and S7 respectively. We can notice that there is a slight increase in the RMSD values after refinement, consistently with what observed in the last CASP10 experiment (11).

An evaluation of the accuracy of LYRA on an independent set of TCR molecules, never used to develop the method, is reported in Supplementary table S8. The accuracy of these models is consistent with the results of the leave-one-out validations. These models are in general of good quality, with the partial exception of some loops. In the two most extreme cases (in which the model RMSD exceeds the leave-one-out mean value by more than two standard deviations), the low sequence similarity to the best template found in our dataset appears to be the cause of the poor model quality. The A2 loop of 4QRP, modeled using the 4P46 template, has a RMSD of 4.53Å and a 29% sequence identity. The B1 loop of 4QOK, that presents a RMSD value of 2.31Å to the native structure, shares only 10% sequence identity with its template 1KB5. These values are consistent with the region-specific RMSD/percent id plots in figure S4. We expect that, as the number of solved structures in our template database will increase, such extreme cases will become less and less frequent.

1. **Database automatic update**

We have implemented a monthly routine to update the template structural database. The sequences of all the new molecules deposited in the last month are scanned with both BCR and TCR HMMs and assigned to the corresponding chain type. Only crystal structures with a resolution better than 3Å are considered. Only a single copy of the complete lymphocyte receptor is retained from each pdb file. Finally we discard all the novel molecules for which both chains are identical (in terms of sequence) to any corresponding chain already present in our structural database and insert in the database all the remaining molecules. The date of the last db update is reported in the footer of the input page.

**References**

1. Chailyan, A., Marcatili, P. and Tramontano, A. (2011) The association of heavy and light chain variable domains in antibodies: implications for antigen specificity. *The FEBS journal*, **278**, 2858-2866.

<http://www.ncbi.nlm.nih.gov/pubmed/21651726>

<http://dx.doi.org/10.1111/j.1742-4658.2011.08207.x>

2. Chailyan, A., Marcatili, P., Cirillo, D. and Tramontano, A. (2011) Structural repertoire of immunoglobulin lambda light chains. *Proteins*, **79**, 1513-1524.

<http://www.ncbi.nlm.nih.gov/pubmed/21365679>

<http://dx.doi.org/10.1002/prot.22979>

3. Kuroda, D., Shirai, H., Kobori, M. and Nakamura, H. (2009) Systematic classification of CDR-L3 in antibodies: implications of the light chain subtypes and the VL-VH interface. *Proteins*, **75**, 139-146.

<http://www.ncbi.nlm.nih.gov/pubmed/18798566>

<http://dx.doi.org/10.1002/prot.22230>

4. Kuroda, D., Shirai, H., Kobori, M. and Nakamura, H. (2008) Structural classification of CDR-H3 revisited: a lesson in antibody modeling. *Proteins*, **73**, 608-620.

<http://www.ncbi.nlm.nih.gov/pubmed/18473362>

<http://dx.doi.org/10.1002/prot.22087>

5. Al-Lazikani, B., Lesk, A.M. and Chothia, C. (1997) Standard conformations for the canonical structures of immunoglobulins. *Journal of molecular biology*, **273**, 927-948.

<http://www.ncbi.nlm.nih.gov/pubmed/9367782>

<http://dx.doi.org/10.1006/jmbi.1997.1354>

6. Shirai, H., Kidera, A. and Nakamura, H. (1996) Structural classification of CDR-H3 in antibodies. *FEBS letters*, **399**, 1-8.

<http://www.ncbi.nlm.nih.gov/pubmed/8980108>

7. Chothia, C., Lesk, A.M., Tramontano, A., Levitt, M., Smith-Gill, S.J., Air, G., Sheriff, S., Padlan, E.A., Davies, D., Tulip, W.R. *et al.* (1989) Conformations of immunoglobulin hypervariable regions. *Nature*, **342**, 877-883.

<http://www.ncbi.nlm.nih.gov/pubmed/2687698>

<http://dx.doi.org/10.1038/342877a0>

8. Chothia, C. and Lesk, A.M. (1987) Canonical structures for the hypervariable regions of immunoglobulins. *Journal of molecular biology*, **196**, 901-917.

<http://www.ncbi.nlm.nih.gov/pubmed/3681981>

9. North, B., Lehmann, A. and Dunbrack, R.L., Jr. (2011) A new clustering of antibody CDR loop conformations. *Journal of molecular biology*, **406**, 228-256.

<http://www.ncbi.nlm.nih.gov/pubmed/21035459>

<http://dx.doi.org/10.1016/j.jmb.2010.10.030>

10. Pedregosa, F., Varoquaux, G., Gramfort, A., Michel, V., Thirion, B., Grisel, O., Blondel, M., Prettenhofer, P., Weiss, R. and Dubourg, V. (2011) Scikit-learn: Machine learning in Python. *The Journal of Machine Learning Research*, **12**, 2825-2830 %@ 1532-4435.

11. Nugent, T., Cozzetto, D. and Jones, D.T. (2014) Evaluation of predictions in the CASP10 model refinement category. *Proteins*, **82 Suppl 2**, 98-111.

<http://www.ncbi.nlm.nih.gov/pubmed/23900810>

<http://dx.doi.org/10.1002/prot.24377>

**Supplementary figures**

**Figure S1:** Modeling accuracy for models generated using the CS (left bars) and sequence identity (right bars) template selection methods. The colored regions of each barplot correspond to the number of models (out of 99) for which a given region (reported on the x-axis) had a C_α_ RMSD to the corresponding region of the solved structure below 1Å (green), between 1 and 2 Å (yellow), between 2 and 5 Å (orange), between 5 and 10 Å (red) or exceeding 10 Å (black). The average RMSD per region is reported above each column.

**Figure S2:** Accuracy of TCR α chain CSs. The box plots describe the accuracy of A1, A2 and A3 loops in models generated using the leave-one-out validation with a 95% identity threshold depending of their CS (as defined in table S1)

**Figure S3:** Accuracy of β chain CSs. The box plots describe the accuracy of B1 and B2 loops in models generated using the leave-one-out validation with a 95% identity threshold depending of their CS (as defined in table S2).

**Figure S4:** Local accuracy as a function of local sequence similarity. A scatterplot of the local accuracy (for every region of α and β chains) in all the models generated in the leave-one-out validation with a similarity threshold of 95% (red dots) and 90% (blue dots) in function of the local sequence similarity with the template used to model the region.

**Supplementary tables**

**Table S1:** Canonical structures of TCR α chain CDRs. For each CDR type (Loop column) and length (Length column) all the CS identified by the clustering algorithm (CS id column) and the PDB code of a representative structure from the cluster (Repres. PDB column) are reported. All CDRs for which no CS can be defined or predicted are labeled as CS0.

| Loop | Length | CS id | Repres. PDB |
| --- | --- | --- | --- |
| Any | Any | 0 |  |
| A1 | 8 | 1 | 2VLR |
|  | 9 | 2 | 3QFJ |
|  |  | 3 | 3TJH |
|  |  | 4 | 3ARB |
|  |  | 5 | 4G8E |
|  | 10 | 6 | 4OZH |
|  |  | 7 | 4JRX |
| A2 | 5 | 1 | 3QIU |
|  |  | 2 | 4OZG |
|  | 6 | 3 | 4L3E |
|  | 7 | 4 | 4P23 |
|  |  | 5 | 3MV8 |
|  |  | 6 | 3REV |
|  |  | 7 | 3SCM |
|  |  | 8 | 4G9F |
| A3 | 9 | 1 | 3SKN |
|  | 10 | 2 | 3QEQ |
|  | 11 | 3 | 4L4V |
|  |  | 4 | 4P46 |
|  | 12 | 5 | 3PL6 |
|  |  | 6 | 3QFJ |
|  | 13 | 7 | 3TF7 |
|  | 14 | 8 | 3HUJ |
|  |  | 9 | 3MV8 |
|  |  | 10 | 2BNQ |
|  | 15 | 11 | 4P4K |
|  |  | 12 | 1MI5 |

**Table S2:** Canonical structures of TCR β chain CDRs. For each CDR type (Loop column) and length (Length column) all the CS identified by the clustering algorithm (CS id column) and the PDB code of a representative structure from the cluster (Repres. PDB column) are reported. All CDRs for which no CS can be defined or predicted are labeled as CS0. No CS is defined for the β CDR3 because of the poor clustering and CS prediction results.

| Loop | Length | CS id | Repres. PDB |
| --- | --- | --- | --- |
| Any | Any | 0 | - |
| B1 | 9 | 1 | 3QEU |
|  |  | 2 | 4MAY |
|  | 10 | 3 | 3O6F |
|  |  | 4 | 4JFF |
| B2 | 17 | 1 | 4JFH |
|  | 18 | 2 | 2IAL |
|  |  | 3 | 3MV8 |
|  | 19 | 4 | 4MAY |
|  |  | 5 | 3QIU |
|  |  | 6 | 1KB5 |
|  | 20 | 7 | 3O6F |
| B3* | Any | 0 | - |

**Table S3:** CS and structural templates for TCR α chain CDR1 and CDR2. For each TCR α functional variable gene present in the IMGT-GENE-DB database, we calculated the predicted CS (A1 CS and A2 Cs columns) together with the number of templates in our structural database with a similar CDR length (Same length columns) and with the same CS (Same CS column). Genes for which the HMM alignment method failed are reported in supplementary table S5.

| TRBV Gene/Allele | A1 CS | Same length | Same CS | A2 CS | Same length | Same CS |
| --- | --- | --- | --- | --- | --- | --- |
| TRAV1*01\|Mus musculus | 5 | 74 | 9 | 3 | 23 | 23 |
| TRAV1*02\|Mus musculus | 5 | 74 | 9 | 3 | 23 | 23 |
| TRAV1-1*01\|Homo sapiens | 5 | 74 | 9 | 3 | 23 | 23 |
| TRAV1-1*02\|Homo sapiens | 5 | 74 | 9 | 3 | 23 | 23 |
| TRAV1-2*01\|Homo sapiens | 5 | 74 | 9 | 3 | 23 | 23 |
| TRAV1-2*02\|Homo sapiens | 5 | 74 | 9 | 3 | 23 | 23 |
| TRAV10*01\|Homo sapiens | 4 | 74 | 8 | 4 | 62 | 29 |
| TRAV10*01\|Mus musculus | 2 | 74 | 38 | 4 | 62 | 29 |
| TRAV10*02\|Mus musculus | 2 | 74 | 38 | 4 | 62 | 29 |
| TRAV10*03\|Mus musculus | 2 | 74 | 38 | 4 | 62 | 29 |
| TRAV10*04\|Mus musculus | 2 | 74 | 38 | 4 | 62 | 29 |
| TRAV10*05\|Mus musculus | 2 | 74 | 38 | 4 | 62 | 29 |
| TRAV10D*01\|Mus musculus | 2 | 74 | 38 | 4 | 62 | 29 |
| TRAV10D*02\|Mus musculus | 2 | 74 | 38 | 4 | 62 | 29 |
| TRAV10N*01\|Mus musculus | 2 | 74 | 38 | 4 | 62 | 29 |
| TRAV11*01\|Mus musculus | 4 | 74 | 8 | 7 | 62 | 9 |
| TRAV11*02\|Mus musculus | 4 | 74 | 8 | 7 | 62 | 9 |
| TRAV11D*01\|Mus musculus | 4 | 74 | 8 | 7 | 62 | 9 |
| TRAV12-1*01\|Homo sapiens | 2 | 74 | 38 | 2 | 17 | 8 |
| TRAV12-1*01\|Mus musculus | 0 | 14 | 0 | 7 | 62 | 9 |
| TRAV12-1*02\|Homo sapiens | 2 | 74 | 38 | 2 | 17 | 8 |
| TRAV12-1*02\|Mus musculus | 7 | 14 | 6 | 7 | 62 | 9 |
| TRAV12-1*03\|Mus musculus | 0 | 14 | 0 | 7 | 62 | 9 |
| TRAV12-1*04\|Mus musculus | 0 | 14 | 0 | 7 | 62 | 9 |
| TRAV12-1*05\|Mus musculus | 0 | 14 | 0 | 7 | 62 | 9 |
| TRAV12-2*01\|Homo sapiens | 2 | 74 | 38 | 3 | 23 | 23 |
| TRAV12-2*01\|Mus musculus | 3 | 74 | 19 | 6 | 62 | 9 |
| TRAV12-2*02\|Homo sapiens | 2 | 74 | 38 | 3 | 23 | 23 |
| TRAV12-2*03\|Homo sapiens | 2 | 74 | 38 | 3 | 23 | 23 |
| TRAV12-3*01\|Homo sapiens | 2 | 74 | 38 | 3 | 23 | 23 |
| TRAV12-3*01\|Mus musculus | 0 | 14 | 0 | 7 | 62 | 9 |
| TRAV12-3*02\|Homo sapiens | 2 | 74 | 38 | 3 | 23 | 23 |
| TRAV12-3*02\|Mus musculus | 0 | 14 | 0 | 6 | 23 | 0 |
| TRAV12-3*03\|Mus musculus | 0 | 14 | 0 | 7 | 62 | 9 |
| TRAV12-3*04\|Mus musculus | 0 | 14 | 0 | 7 | 62 | 9 |
| TRAV12D-1*01\|Mus musculus | 7 | 14 | 6 | 7 | 62 | 9 |
| TRAV12D-1*02\|Mus musculus | 7 | 14 | 6 | 7 | 62 | 9 |
| TRAV12D-1*03\|Mus musculus | 0 | 0 | 0 | 7 | 62 | 9 |
| TRAV12D-1*04\|Mus musculus | 7 | 14 | 6 | 7 | 62 | 9 |
| TRAV12D-1*05\|Mus musculus | 7 | 14 | 6 | 7 | 62 | 9 |
| TRAV12D-2*01\|Mus musculus | 3 | 74 | 19 | 6 | 62 | 9 |
| TRAV12D-2*02\|Mus musculus | 3 | 74 | 19 | 6 | 62 | 9 |
| TRAV12D-2*03\|Mus musculus | 3 | 74 | 19 | 6 | 62 | 9 |
| TRAV12D-2*04\|Mus musculus | 3 | 74 | 19 | 6 | 62 | 9 |
| TRAV12D-2*05\|Mus musculus | 3 | 74 | 19 | 6 | 62 | 9 |
| TRAV12D-3*01\|Mus musculus | 0 | 14 | 0 | 6 | 62 | 9 |
| TRAV12D-3*02\|Mus musculus | 0 | 14 | 0 | 7 | 62 | 9 |
| TRAV12D-3*03\|Mus musculus | 0 | 14 | 0 | 7 | 62 | 9 |
| TRAV12N-1*01\|Mus musculus | 0 | 14 | 0 | 7 | 62 | 9 |
| TRAV12N-2*01\|Mus musculus | 3 | 74 | 19 | 6 | 62 | 9 |
| TRAV12N-3*01\|Mus musculus | 0 | 14 | 0 | 6 | 62 | 9 |
| TRAV13-1*01\|Homo sapiens | 2 | 74 | 38 | 4 | 62 | 29 |
| TRAV13-1*01\|Mus musculus | 1 | 19 | 19 | 1 | 17 | 9 |
| TRAV13-1*02\|Homo sapiens | 2 | 74 | 38 | 4 | 62 | 29 |
| TRAV13-1*03\|Homo sapiens | 2 | 74 | 38 | 4 | 62 | 29 |
| TRAV13-2*01\|Homo sapiens | 2 | 74 | 38 | 4 | 62 | 29 |
| TRAV13-2*01\|Mus musculus | 1 | 19 | 19 | 1 | 17 | 9 |
| TRAV13-2*02\|Homo sapiens | 2 | 74 | 38 | 4 | 62 | 29 |
| TRAV13-2*02\|Mus musculus | 1 | 19 | 19 | 1 | 17 | 9 |
| TRAV13-3*01\|Mus musculus | 1 | 19 | 19 | 3 | 23 | 23 |
| TRAV13-3*02\|Mus musculus | 1 | 19 | 19 | 3 | 23 | 23 |
| TRAV13-4/DV7*01\|Mus musculus | 1 | 19 | 19 | 1 | 17 | 9 |
| TRAV13-4/DV7*02\|Mus musculus | 1 | 19 | 19 | 3 | 17 | 0 |
| TRAV13-4/DV7*03\|Mus musculus | 1 | 19 | 19 | 1 | 17 | 9 |
| TRAV13-5*01\|Mus musculus | 1 | 19 | 19 | 4 | 17 | 0 |
| TRAV13D-1*01\|Mus musculus | 1 | 19 | 19 | 1 | 17 | 9 |
| TRAV13D-1*02\|Mus musculus | 1 | 19 | 19 | 1 | 17 | 9 |
| TRAV13D-1*03\|Mus musculus | 0 | 0 | 0 | 1 | 17 | 9 |
| TRAV13D-2*01\|Mus musculus | 1 | 19 | 19 | 1 | 17 | 9 |
| TRAV13D-2*02\|Mus musculus | 1 | 19 | 19 | 1 | 17 | 9 |
| TRAV13D-3*01\|Mus musculus | 1 | 19 | 19 | 3 | 23 | 23 |
| TRAV13D-4*01\|Mus musculus | 1 | 19 | 19 | 3 | 17 | 0 |
| TRAV13D-4*02\|Mus musculus | 1 | 19 | 19 | 3 | 17 | 0 |
| TRAV13D-4*03\|Mus musculus | 1 | 19 | 19 | 1 | 17 | 9 |
| TRAV13N-1*01\|Mus musculus | 1 | 19 | 19 | 1 | 17 | 9 |
| TRAV13N-2*01\|Mus musculus | 1 | 19 | 19 | 1 | 17 | 9 |
| TRAV13N-4*01\|Mus musculus | 1 | 19 | 19 | 3 | 17 | 0 |
| TRAV14-1*01\|Mus musculus | 2 | 74 | 38 | 4 | 62 | 29 |
| TRAV14-1*02\|Mus musculus | 2 | 74 | 38 | 4 | 62 | 29 |
| TRAV14-1*03\|Mus musculus | 2 | 74 | 38 | 4 | 62 | 29 |
| TRAV14-2*01\|Mus musculus | 2 | 74 | 38 | 4 | 62 | 29 |
| TRAV14-2*02\|Mus musculus | 2 | 74 | 38 | 4 | 62 | 29 |
| TRAV14-2*03\|Mus musculus | 2 | 74 | 38 | 4 | 62 | 29 |
| TRAV14-3*01\|Mus musculus | 2 | 74 | 38 | 4 | 62 | 29 |
| TRAV14-3*02\|Mus musculus | 2 | 74 | 38 | 4 | 62 | 29 |
| TRAV14-3*03\|Mus musculus | 2 | 74 | 38 | 4 | 62 | 29 |
| TRAV14/DV4*01\|Homo sapiens | 7 | 14 | 6 | 8 | 5 | 5 |
| TRAV14/DV4*02\|Homo sapiens | 7 | 14 | 6 | 8 | 5 | 5 |
| TRAV14/DV4*03\|Homo sapiens | 7 | 14 | 6 | 8 | 5 | 5 |
| TRAV14/DV4*04\|Homo sapiens | 7 | 14 | 6 | 8 | 5 | 5 |
| TRAV14D-1*01\|Mus musculus | 2 | 74 | 38 | 4 | 62 | 29 |
| TRAV14D-1*02\|Mus musculus | 2 | 74 | 38 | 4 | 62 | 29 |
| TRAV14D-2*01\|Mus musculus | 2 | 74 | 38 | 4 | 62 | 29 |
| TRAV14D-2*02\|Mus musculus | 0 | 0 | 0 | 4 | 62 | 29 |
| TRAV14D-2*03\|Mus musculus | 2 | 74 | 38 | 4 | 62 | 29 |
| TRAV14D-3/DV8*01\|Mus musculus | 2 | 74 | 38 | 4 | 62 | 29 |
| TRAV14D-3/DV8*02\|Mus musculus | 2 | 74 | 38 | 4 | 62 | 29 |
| TRAV14D-3/DV8*03\|Mus musculus | 2 | 74 | 38 | 4 | 62 | 29 |
| TRAV14D-3/DV8*04\|Mus musculus | 2 | 74 | 38 | 4 | 62 | 29 |
| TRAV14D-3/DV8*05\|Mus musculus | 2 | 74 | 38 | 4 | 62 | 29 |
| TRAV14D-3/DV8*06\|Mus musculus | 2 | 74 | 38 | 4 | 62 | 29 |
| TRAV14D-3/DV8*07\|Mus musculus | 2 | 74 | 38 | 4 | 62 | 29 |
| TRAV14D-3/DV8*08\|Mus musculus | 2 | 74 | 38 | 4 | 62 | 29 |
| TRAV14N-1*01\|Mus musculus | 2 | 74 | 38 | 4 | 62 | 29 |
| TRAV14N-2*01\|Mus musculus | 2 | 74 | 38 | 4 | 62 | 29 |
| TRAV14N-3*01\|Mus musculus | 2 | 74 | 38 | 4 | 62 | 29 |
| TRAV15-1/DV6-1*01\|Mus musculus | 7 | 14 | 6 | 4 | 5 | 0 |
| TRAV15-1/DV6-1*02\|Mus musculus | 7 | 14 | 6 | 4 | 5 | 0 |
| TRAV15-2/DV6-2*01\|Mus musculus | 7 | 14 | 6 | 4 | 62 | 29 |
| TRAV15-2/DV6-2*02\|Mus musculus | 7 | 14 | 6 | 4 | 62 | 29 |
| TRAV15D-1/DV6D-1*01\|Mus musculus | 7 | 14 | 6 | 4 | 62 | 29 |
| TRAV15D-1/DV6D-1*02\|Mus musculus | 7 | 14 | 6 | 4 | 62 | 29 |
| TRAV15D-1/DV6D-1*03\|Mus musculus | 7 | 14 | 6 | 4 | 5 | 0 |
| TRAV15D-1/DV6D-1*04\|Mus musculus | 7 | 14 | 6 | 4 | 62 | 29 |
| TRAV15D-1/DV6D-1*05\|Mus musculus | 7 | 14 | 6 | 8 | 62 | 0 |
| TRAV15D-1/DV6D-1*06\|Mus musculus | 7 | 14 | 6 | 4 | 62 | 29 |
| TRAV15D-2/DV6D-2*01\|Mus musculus | 7 | 14 | 6 | 4 | 62 | 29 |
| TRAV15D-2/DV6D-2*02\|Mus musculus | 7 | 14 | 6 | 4 | 62 | 29 |
| TRAV15D-2/DV6D-2*03\|Mus musculus | 7 | 14 | 6 | 4 | 62 | 29 |
| TRAV15D-2/DV6D-2*04\|Mus musculus | 7 | 14 | 6 | 4 | 62 | 29 |
| TRAV15D-2/DV6D-2*05\|Mus musculus | 7 | 14 | 6 | 4 | 62 | 29 |
| TRAV15N-1*01\|Mus musculus | 7 | 14 | 6 | 4 | 62 | 29 |
| TRAV15N-2*01\|Mus musculus | 7 | 14 | 6 | 4 | 62 | 29 |
| TRAV16*01\|Homo sapiens | 3 | 74 | 19 | 4 | 0 | 0 |
| TRAV16*01\|Mus musculus | 7 | 14 | 6 | 8 | 5 | 5 |
| TRAV16*02\|Mus musculus | 7 | 14 | 6 | 8 | 5 | 5 |
| TRAV16*03\|Mus musculus | 7 | 14 | 6 | 8 | 5 | 5 |
| TRAV16*04\|Mus musculus | 7 | 14 | 6 | 8 | 5 | 5 |
| TRAV16*05\|Mus musculus | 7 | 14 | 6 | 8 | 5 | 5 |
| TRAV16D/DV11*01\|Mus musculus | 7 | 14 | 6 | 8 | 5 | 5 |
| TRAV16D/DV11*02\|Mus musculus | 7 | 14 | 6 | 8 | 5 | 5 |
| TRAV16D/DV11*03\|Mus musculus | 7 | 14 | 6 | 8 | 5 | 5 |
| TRAV16N*01\|Mus musculus | 7 | 14 | 6 | 8 | 5 | 5 |
| TRAV17*01\|Homo sapiens | 1 | 19 | 19 | 4 | 62 | 29 |
| TRAV17*01\|Mus musculus | 3 | 74 | 19 | 6 | 62 | 9 |
| TRAV17*02\|Mus musculus | 3 | 74 | 19 | 6 | 62 | 9 |
| TRAV18*01\|Homo sapiens | 3 | 74 | 19 | 0 | 23 | 0 |
| TRAV19*01\|Homo sapiens | 7 | 14 | 6 | 8 | 5 | 5 |
| TRAV19*01\|Mus musculus | 2 | 74 | 38 | 4 | 62 | 29 |
| TRAV19*03\|Mus musculus | 2 | 74 | 38 | 4 | 62 | 29 |
| TRAV2*01\|Homo sapiens | 0 | 74 | 0 | 0 | 0 | 0 |
| TRAV2*01\|Mus musculus | 6 | 14 | 8 | 2 | 17 | 8 |
| TRAV2*02\|Homo sapiens | 1 | 74 | 0 | 0 | 0 | 0 |
| TRAV20*01\|Homo sapiens | 5 | 74 | 9 | 5 | 62 | 15 |
| TRAV20*02\|Homo sapiens | 5 | 74 | 9 | 5 | 62 | 15 |
| TRAV20*03\|Homo sapiens | 5 | 74 | 9 | 5 | 62 | 15 |
| TRAV20*04\|Homo sapiens | 5 | 74 | 9 | 5 | 62 | 15 |
| TRAV21*01\|Homo sapiens | 2 | 74 | 38 | 4 | 62 | 29 |
| TRAV21*02\|Homo sapiens | 2 | 74 | 38 | 4 | 62 | 29 |
| TRAV21/DV12*01\|Mus musculus | 6 | 14 | 8 | 2 | 17 | 8 |
| TRAV21/DV12*02\|Mus musculus | 6 | 14 | 8 | 2 | 17 | 8 |
| TRAV22*01\|Homo sapiens | 1 | 19 | 19 | 1 | 17 | 9 |
| TRAV23/DV6*01\|Homo sapiens | 2 | 74 | 38 | 4 | 62 | 29 |
| TRAV23/DV6*02\|Homo sapiens | 2 | 74 | 38 | 4 | 62 | 29 |
| TRAV23/DV6*03\|Homo sapiens | 2 | 74 | 38 | 4 | 62 | 29 |
| TRAV23/DV6*04\|Homo sapiens | 2 | 74 | 38 | 4 | 62 | 29 |
| TRAV24*01\|Homo sapiens | 2 | 74 | 38 | 5 | 62 | 15 |
| TRAV24*02\|Homo sapiens | 2 | 74 | 38 | 5 | 62 | 15 |
| TRAV25*01\|Homo sapiens | 1 | 19 | 19 | 0 | 23 | 0 |
| TRAV26-1*01\|Homo sapiens | 6 | 14 | 8 | 2 | 17 | 8 |
| TRAV26-1*02\|Homo sapiens | 6 | 14 | 8 | 2 | 17 | 8 |
| TRAV26-1*03\|Homo sapiens | 6 | 14 | 8 | 2 | 17 | 8 |
| TRAV26-2*01\|Homo sapiens | 6 | 14 | 8 | 2 | 17 | 8 |
| TRAV26-2*02\|Homo sapiens | 6 | 14 | 8 | 2 | 17 | 8 |
| TRAV27*01\|Homo sapiens | 1 | 19 | 19 | 5 | 62 | 15 |
| TRAV27*02\|Homo sapiens | 1 | 19 | 19 | 5 | 62 | 15 |
| TRAV27*03\|Homo sapiens | 1 | 19 | 19 | 5 | 62 | 15 |
| TRAV29/DV5*01\|Homo sapiens | 2 | 74 | 38 | 4 | 62 | 29 |
| TRAV29/DV5*02\|Homo sapiens | 2 | 74 | 38 | 4 | 62 | 29 |
| TRAV3*01\|Homo sapiens | 3 | 74 | 19 | 6 | 62 | 9 |
| TRAV3-1*01\|Mus musculus | 2 | 74 | 38 | 4 | 62 | 29 |
| TRAV3-1*02\|Mus musculus | 2 | 74 | 38 | 4 | 62 | 29 |
| TRAV3-3*01\|Mus musculus | 2 | 74 | 38 | 4 | 62 | 29 |
| TRAV3-4*01\|Mus musculus | 2 | 74 | 38 | 4 | 62 | 29 |
| TRAV30*01\|Homo sapiens | 1 | 19 | 19 | 5 | 62 | 15 |
| TRAV30*02\|Homo sapiens | 1 | 19 | 19 | 5 | 62 | 15 |
| TRAV30*03\|Homo sapiens | 1 | 19 | 19 | 5 | 62 | 15 |
| TRAV30*04\|Homo sapiens | 1 | 19 | 19 | 5 | 62 | 15 |
| TRAV34*01\|Homo sapiens | 1 | 19 | 19 | 4 | 62 | 29 |
| TRAV35*01\|Homo sapiens | 1 | 19 | 19 | 7 | 62 | 9 |
| TRAV35*02\|Homo sapiens | 1 | 19 | 19 | 7 | 62 | 9 |
| TRAV36/DV7*01\|Homo sapiens | 2 | 74 | 38 | 4 | 62 | 29 |
| TRAV36/DV7*02\|Homo sapiens | 2 | 74 | 38 | 4 | 62 | 29 |
| TRAV36/DV7*03\|Homo sapiens | 2 | 74 | 38 | 4 | 62 | 29 |
| TRAV36/DV7*04\|Homo sapiens | 2 | 74 | 38 | 4 | 62 | 29 |
| TRAV38-1*01\|Homo sapiens | 7 | 14 | 6 | 8 | 5 | 5 |
| TRAV38-1*02\|Homo sapiens | 7 | 14 | 6 | 8 | 5 | 5 |
| TRAV38-1*03\|Homo sapiens | 7 | 14 | 6 | 8 | 5 | 5 |
| TRAV38-1*04\|Homo sapiens | 7 | 14 | 6 | 8 | 5 | 5 |
| TRAV38-2/DV8*01\|Homo sapiens | 7 | 14 | 6 | 8 | 5 | 5 |
| TRAV39*01\|Homo sapiens | 1 | 19 | 19 | 5 | 62 | 15 |
| TRAV3D-3*01\|Mus musculus | 2 | 74 | 38 | 4 | 62 | 29 |
| TRAV3D-3*02\|Mus musculus | 2 | 74 | 38 | 4 | 62 | 29 |
| TRAV3N-3*01\|Mus musculus | 2 | 74 | 38 | 4 | 62 | 29 |
| TRAV4*01\|Homo sapiens | 6 | 14 | 8 | 2 | 17 | 8 |
| TRAV4-2*01\|Mus musculus | 1 | 19 | 19 | 1 | 17 | 9 |
| TRAV4-2*02\|Mus musculus | 1 | 19 | 19 | 1 | 17 | 9 |
| TRAV4-3*01\|Mus musculus | 1 | 19 | 19 | 1 | 17 | 9 |
| TRAV4-3*02\|Mus musculus | 1 | 19 | 19 | 1 | 17 | 9 |
| TRAV4-4/DV10*01\|Mus musculus | 1 | 19 | 19 | 1 | 17 | 9 |
| TRAV40*01\|Homo sapiens | 3 | 74 | 19 | 0 | 0 | 0 |
| TRAV41*01\|Homo sapiens | 1 | 19 | 19 | 4 | 17 | 0 |
| TRAV4D-3*01\|Mus musculus | 1 | 19 | 19 | 1 | 17 | 9 |
| TRAV4D-3*02\|Mus musculus | 1 | 19 | 19 | 1 | 17 | 9 |
| TRAV4D-3*03\|Mus musculus | 1 | 19 | 19 | 1 | 17 | 9 |
| TRAV4D-3*04\|Mus musculus | 1 | 19 | 19 | 1 | 17 | 9 |
| TRAV4D-4*01\|Mus musculus | 1 | 19 | 19 | 1 | 17 | 9 |
| TRAV4D-4*02\|Mus musculus | 1 | 19 | 19 | 1 | 17 | 9 |
| TRAV4D-4*03\|Mus musculus | 1 | 19 | 19 | 1 | 17 | 9 |
| TRAV4D-4*04\|Mus musculus | 1 | 19 | 19 | 1 | 17 | 9 |
| TRAV4N-3*01\|Mus musculus | 1 | 19 | 19 | 1 | 17 | 9 |
| TRAV4N-4*01\|Mus musculus | 1 | 19 | 19 | 1 | 17 | 9 |
| TRAV5*01\|Homo sapiens | 2 | 74 | 38 | 4 | 62 | 29 |
| TRAV5-1*01\|Mus musculus | 2 | 74 | 38 | 4 | 62 | 29 |
| TRAV5D-4*01\|Mus musculus | 2 | 74 | 38 | 4 | 62 | 29 |
| TRAV5D-4*02\|Mus musculus | 2 | 74 | 38 | 4 | 62 | 29 |
| TRAV5D-4*03\|Mus musculus | 2 | 74 | 38 | 4 | 62 | 29 |
| TRAV5D-4*04\|Mus musculus | 2 | 74 | 38 | 4 | 62 | 29 |
| TRAV5D-4*05\|Mus musculus | 2 | 74 | 38 | 4 | 62 | 29 |
| TRAV5N-4*01\|Mus musculus | 2 | 74 | 38 | 4 | 62 | 29 |
| TRAV6*01\|Homo sapiens | 0 | 74 | 0 | 4 | 62 | 29 |
| TRAV6*02\|Homo sapiens | 0 | 74 | 0 | 4 | 62 | 29 |
| TRAV6*03\|Homo sapiens | 0 | 74 | 0 | 4 | 62 | 29 |
| TRAV6*04\|Homo sapiens | 0 | 74 | 0 | 4 | 62 | 29 |
| TRAV6*05\|Homo sapiens | 0 | 74 | 0 | 4 | 62 | 29 |
| TRAV6*06\|Homo sapiens | 1 | 74 | 0 | 4 | 62 | 29 |
| TRAV6-1*01\|Mus musculus | 3 | 74 | 19 | 7 | 62 | 9 |
| TRAV6-1*02\|Mus musculus | 3 | 74 | 19 | 7 | 62 | 9 |
| TRAV6-2*01\|Mus musculus | 3 | 74 | 19 | 7 | 62 | 9 |
| TRAV6-2*02\|Mus musculus | 3 | 74 | 19 | 7 | 62 | 9 |
| TRAV6-2*03\|Mus musculus | 3 | 74 | 19 | 7 | 62 | 9 |
| TRAV6-3*01\|Mus musculus | 3 | 74 | 19 | 7 | 62 | 9 |
| TRAV6-3*02\|Mus musculus | 3 | 74 | 19 | 7 | 62 | 9 |
| TRAV6-4*01\|Mus musculus | 3 | 74 | 19 | 7 | 62 | 9 |
| TRAV6-4*02\|Mus musculus | 3 | 74 | 19 | 7 | 62 | 9 |
| TRAV6-4*03\|Mus musculus | 3 | 74 | 19 | 7 | 62 | 9 |
| TRAV6-5*01\|Mus musculus | 3 | 74 | 19 | 5 | 62 | 15 |
| TRAV6-5*02\|Mus musculus | 3 | 74 | 19 | 5 | 62 | 15 |
| TRAV6-5*03\|Mus musculus | 3 | 74 | 19 | 5 | 62 | 15 |
| TRAV6-5*04\|Mus musculus | 3 | 74 | 19 | 5 | 62 | 15 |
| TRAV6-6*01\|Mus musculus | 0 | 0 | 0 | 5 | 62 | 15 |
| TRAV6-6*02\|Mus musculus | 0 | 0 | 0 | 5 | 62 | 15 |
| TRAV6-7/DV9*01\|Mus musculus | 3 | 74 | 19 | 5 | 62 | 15 |
| TRAV6-7/DV9*02\|Mus musculus | 3 | 74 | 19 | 5 | 62 | 15 |
| TRAV6-7/DV9*03\|Mus musculus | 3 | 74 | 19 | 5 | 62 | 15 |
| TRAV6-7/DV9*04\|Mus musculus | 3 | 74 | 19 | 5 | 62 | 15 |
| TRAV6-7/DV9*06\|Mus musculus | 3 | 74 | 19 | 5 | 62 | 15 |
| TRAV6-7/DV9*07\|Mus musculus | 3 | 74 | 19 | 5 | 62 | 15 |
| TRAV6-7/DV9*08\|Mus musculus | 3 | 0 | 0 | 5 | 62 | 15 |
| TRAV6D-3*01\|Mus musculus | 3 | 74 | 19 | 7 | 62 | 9 |
| TRAV6D-3*02\|Mus musculus | 3 | 74 | 19 | 7 | 62 | 9 |
| TRAV6D-4*01\|Mus musculus | 3 | 74 | 19 | 7 | 62 | 9 |
| TRAV6D-5*01\|Mus musculus | 3 | 74 | 19 | 5 | 62 | 15 |
| TRAV6D-6*01\|Mus musculus | 0 | 0 | 0 | 5 | 62 | 15 |
| TRAV6D-6*02\|Mus musculus | 0 | 0 | 0 | 5 | 62 | 15 |
| TRAV6D-6*03\|Mus musculus | 0 | 0 | 0 | 5 | 62 | 15 |
| TRAV6D-6*04\|Mus musculus | 0 | 0 | 0 | 5 | 62 | 15 |
| TRAV6D-6*05\|Mus musculus | 0 | 0 | 0 | 5 | 62 | 15 |
| TRAV6D-7*01\|Mus musculus | 3 | 74 | 19 | 5 | 62 | 15 |
| TRAV6D-7*02\|Mus musculus | 3 | 74 | 19 | 5 | 62 | 15 |
| TRAV6D-7*03\|Mus musculus | 3 | 19 | 0 | 5 | 62 | 15 |
| TRAV6D-7*04\|Mus musculus | 3 | 74 | 19 | 5 | 62 | 15 |
| TRAV6N-5*01\|Mus musculus | 3 | 74 | 19 | 5 | 62 | 15 |
| TRAV6N-6*01\|Mus musculus | 0 | 0 | 0 | 5 | 62 | 15 |
| TRAV6N-7*01\|Mus musculus | 3 | 74 | 19 | 5 | 62 | 15 |
| TRAV7*01\|Homo sapiens | 1 | 74 | 0 | 4 | 62 | 29 |
| TRAV7-1*01\|Mus musculus | 2 | 74 | 38 | 3 | 23 | 23 |
| TRAV7-2*01\|Mus musculus | 2 | 74 | 38 | 3 | 23 | 23 |
| TRAV7-2*02\|Mus musculus | 2 | 74 | 38 | 3 | 23 | 23 |
| TRAV7-3*01\|Mus musculus | 2 | 74 | 38 | 3 | 23 | 23 |
| TRAV7-3*02\|Mus musculus | 0 | 0 | 0 | 3 | 23 | 23 |
| TRAV7-3*03\|Mus musculus | 2 | 74 | 38 | 3 | 23 | 23 |
| TRAV7-3*04\|Mus musculus | 2 | 74 | 38 | 3 | 23 | 23 |
| TRAV7-4*01\|Mus musculus | 2 | 74 | 38 | 3 | 23 | 23 |
| TRAV7-4*02\|Mus musculus | 2 | 74 | 38 | 3 | 23 | 23 |
| TRAV7-5*01\|Mus musculus | 2 | 74 | 38 | 3 | 23 | 23 |
| TRAV7-5*02\|Mus musculus | 0 | 0 | 0 | 3 | 23 | 23 |
| TRAV7-5*03\|Mus musculus | 0 | 0 | 0 | 3 | 23 | 23 |
| TRAV7-6*01\|Mus musculus | 2 | 74 | 38 | 3 | 23 | 23 |
| TRAV7-6*02\|Mus musculus | 2 | 74 | 38 | 3 | 23 | 23 |
| TRAV7D-2*01\|Mus musculus | 2 | 74 | 38 | 3 | 23 | 23 |
| TRAV7D-2*02\|Mus musculus | 2 | 74 | 38 | 3 | 23 | 23 |
| TRAV7D-3*01\|Mus musculus | 2 | 74 | 38 | 3 | 23 | 23 |
| TRAV7D-3*02\|Mus musculus | 2 | 74 | 38 | 3 | 23 | 23 |
| TRAV7D-4*01\|Mus musculus | 2 | 74 | 38 | 3 | 23 | 23 |
| TRAV7D-4*02\|Mus musculus | 2 | 74 | 38 | 3 | 23 | 23 |
| TRAV7D-4*03\|Mus musculus | 2 | 74 | 38 | 3 | 23 | 23 |
| TRAV7D-5*01\|Mus musculus | 2 | 74 | 38 | 3 | 23 | 23 |
| TRAV7D-6*01\|Mus musculus | 2 | 74 | 38 | 3 | 23 | 23 |
| TRAV7D-6*02\|Mus musculus | 2 | 74 | 38 | 3 | 23 | 23 |
| TRAV7N-4*01\|Mus musculus | 2 | 74 | 38 | 3 | 23 | 23 |
| TRAV7N-5*01\|Mus musculus | 2 | 74 | 38 | 3 | 23 | 23 |
| TRAV7N-6*01\|Mus musculus | 2 | 74 | 38 | 3 | 23 | 23 |
| TRAV8-1*01\|Homo sapiens | 3 | 74 | 19 | 6 | 62 | 9 |
| TRAV8-1*01\|Mus musculus | 1 | 19 | 19 | 4 | 62 | 29 |
| TRAV8-1*02\|Homo sapiens | 3 | 74 | 19 | 6 | 62 | 9 |
| TRAV8-1*02\|Mus musculus | 0 | 0 | 0 | 4 | 23 | 0 |
| TRAV8-1*03\|Mus musculus | 1 | 19 | 19 | 4 | 62 | 29 |
| TRAV8-2*01\|Homo sapiens | 3 | 74 | 19 | 4 | 62 | 29 |
| TRAV8-2*01\|Mus musculus | 1 | 19 | 19 | 4 | 62 | 29 |
| TRAV8-2*02\|Homo sapiens | 3 | 74 | 19 | 4 | 62 | 29 |
| TRAV8-3*01\|Homo sapiens | 3 | 74 | 19 | 6 | 62 | 9 |
| TRAV8-3*02\|Homo sapiens | 3 | 74 | 19 | 6 | 62 | 9 |
| TRAV8-3*03\|Homo sapiens | 3 | 74 | 19 | 6 | 62 | 9 |
| TRAV8-4*01\|Homo sapiens | 3 | 74 | 19 | 4 | 62 | 29 |
| TRAV8-4*02\|Homo sapiens | 3 | 74 | 19 | 4 | 62 | 29 |
| TRAV8-4*03\|Homo sapiens | 3 | 74 | 19 | 4 | 62 | 29 |
| TRAV8-4*04\|Homo sapiens | 3 | 74 | 19 | 4 | 62 | 29 |
| TRAV8-4*05\|Homo sapiens | 3 | 74 | 19 | 4 | 62 | 29 |
| TRAV8-4*06\|Homo sapiens | 0 | 0 | 0 | 4 | 62 | 29 |
| TRAV8-4*07\|Homo sapiens | 0 | 0 | 0 | 4 | 62 | 29 |
| TRAV8-6*01\|Homo sapiens | 3 | 74 | 19 | 4 | 62 | 29 |
| TRAV8-6*02\|Homo sapiens | 3 | 74 | 19 | 4 | 62 | 29 |
| TRAV8D-1*01\|Mus musculus | 1 | 19 | 19 | 4 | 62 | 29 |
| TRAV8D-1*02\|Mus musculus | 1 | 19 | 19 | 4 | 62 | 29 |
| TRAV8D-2*01\|Mus musculus | 1 | 19 | 19 | 4 | 62 | 29 |
| TRAV8D-2*02\|Mus musculus | 1 | 19 | 19 | 4 | 62 | 29 |
| TRAV8D-2*03\|Mus musculus | 1 | 19 | 19 | 4 | 62 | 29 |
| TRAV8N-2*01\|Mus musculus | 1 | 19 | 19 | 4 | 62 | 29 |
| TRAV9-1*01\|Homo sapiens | 3 | 74 | 19 | 4 | 62 | 29 |
| TRAV9-1*01\|Mus musculus | 3 | 74 | 19 | 6 | 62 | 9 |
| TRAV9-1*02\|Mus musculus | 3 | 74 | 19 | 6 | 62 | 9 |
| TRAV9-2*01\|Homo sapiens | 3 | 74 | 19 | 5 | 62 | 15 |
| TRAV9-2*01\|Mus musculus | 3 | 74 | 19 | 6 | 62 | 9 |
| TRAV9-2*02\|Homo sapiens | 3 | 74 | 19 | 4 | 62 | 29 |
| TRAV9-2*03\|Homo sapiens | 3 | 74 | 19 | 4 | 62 | 29 |
| TRAV9-2*04\|Homo sapiens | 3 | 74 | 19 | 5 | 62 | 15 |
| TRAV9-3*01\|Mus musculus | 3 | 74 | 19 | 6 | 62 | 9 |
| TRAV9-3*02\|Mus musculus | 3 | 74 | 19 | 6 | 62 | 9 |
| TRAV9-3*03\|Mus musculus | 3 | 74 | 19 | 6 | 62 | 9 |
| TRAV9-4*01\|Mus musculus | 3 | 74 | 19 | 6 | 62 | 9 |
| TRAV9D-1*01\|Mus musculus | 3 | 74 | 19 | 6 | 62 | 9 |
| TRAV9D-1*02\|Mus musculus | 3 | 74 | 19 | 6 | 62 | 9 |
| TRAV9D-2*01\|Mus musculus | 3 | 74 | 19 | 6 | 62 | 9 |
| TRAV9D-2*02\|Mus musculus | 3 | 74 | 19 | 6 | 62 | 9 |
| TRAV9D-2*03\|Mus musculus | 3 | 74 | 19 | 6 | 62 | 9 |
| TRAV9D-3*01\|Mus musculus | 3 | 74 | 19 | 6 | 62 | 9 |
| TRAV9D-3*02\|Mus musculus | 3 | 74 | 19 | 6 | 62 | 9 |
| TRAV9D-4*01\|Mus musculus | 3 | 74 | 19 | 6 | 62 | 9 |
| TRAV9D-4*03\|Mus musculus | 3 | 0 | 0 | 6 | 62 | 9 |
| TRAV9D-4*04\|Mus musculus | 3 | 74 | 19 | 6 | 62 | 9 |
| TRAV9N-2*01\|Mus musculus | 3 | 74 | 19 | 6 | 62 | 9 |
| TRAV9N-3*01\|Mus musculus | 3 | 74 | 19 | 6 | 62 | 9 |
| TRAV9N-4*01\|Mus musculus | 3 | 74 | 19 | 6 | 62 | 9 |

**Table S4:** CS and structural templates for TCR β chain CDR1 and CDR2. For each TCR β functional variable gene present in the IMGT-GENE-DB database, we calculated the predicted CS (B1 CS and B2 CS columns) together with the number of templates in our structural database with a similar CDR length (Same length columns) and with the same CS (Same CS column). Genes for which the HMM alignment method failed are reported in supplementary table S5.

| TRBV Gene/Allele | B1 CS | Same length | Same CS | B2 CS | Same length | Same CS |
| --- | --- | --- | --- | --- | --- | --- |
| TRBV1*01\|Canis lupus familiaris | 3 | 9 | 6 | 0 | 0 | 0 |
| TRBV1*01\|Mus musculus | 3 | 9 | 6 | 2 | 20 | 6 |
| TRBV1*02\|Mus musculus | 3 | 9 | 6 | 2 | 20 | 6 |
| TRBV10*01\|Canis lupus familiaris | 1 | 105 | 78 | 75 | 86 | 2 |
| TRBV10-1*01\|Homo sapiens | 1 | 105 | 78 | 75 | 86 | 2 |
| TRBV10-1*01\|Macaca mulatta | 1 | 105 | 78 | 75 | 86 | 2 |
| TRBV10-1*02\|Homo sapiens | 1 | 105 | 78 | 75 | 86 | 2 |
| TRBV10-2*01\|Homo sapiens | 1 | 105 | 78 | 75 | 86 | 2 |
| TRBV10-2*01\|Macaca mulatta | 1 | 105 | 78 | 75 | 86 | 2 |
| TRBV10-2*02\|Homo sapiens | 1 | 105 | 78 | 75 | 86 | 2 |
| TRBV10-3*01\|Homo sapiens | 1 | 105 | 78 | 75 | 86 | 2 |
| TRBV10-3*01\|Macaca mulatta | 1 | 105 | 78 | 75 | 86 | 2 |
| TRBV10-3*02\|Homo sapiens | 1 | 105 | 78 | 75 | 86 | 2 |
| TRBV10-3*03\|Homo sapiens | 1 | 105 | 78 | 75 | 86 | 2 |
| TRBV10-3*04\|Homo sapiens | 1 | 105 | 78 | 75 | 86 | 2 |
| TRBV11-1*01\|Homo sapiens | 2 | 105 | 27 | 12 | 20 | 4 |
| TRBV11-1*01\|Macaca mulatta | 2 | 105 | 27 | 12 | 20 | 4 |
| TRBV11-2*01\|Homo sapiens | 2 | 105 | 27 | 12 | 20 | 4 |
| TRBV11-2*01\|Macaca mulatta | 2 | 105 | 27 | 12 | 20 | 4 |
| TRBV11-2*02\|Homo sapiens | 2 | 105 | 27 | 12 | 20 | 4 |
| TRBV11-2*03\|Homo sapiens | 2 | 105 | 27 | 12 | 20 | 4 |
| TRBV11-3*01\|Homo sapiens | 2 | 105 | 27 | 12 | 20 | 4 |
| TRBV11-3*01\|Macaca mulatta | 2 | 105 | 27 | 12 | 20 | 4 |
| TRBV11-3*02\|Homo sapiens | 2 | 105 | 27 | 12 | 20 | 4 |
| TRBV11-3*03\|Homo sapiens | 2 | 105 | 27 | 12 | 20 | 4 |
| TRBV12-1*01\|Macaca mulatta | 2 | 105 | 27 | 12 | 20 | 4 |
| TRBV12-1*01\|Mus musculus | 2 | 105 | 27 | 10 | 86 | 3 |
| TRBV12-1*02\|Mus musculus | 2 | 105 | 27 | 10 | 86 | 3 |
| TRBV12-2*01\|Canis lupus familiaris | 2 | 105 | 27 | 12 | 20 | 4 |
| TRBV12-2*01\|Macaca mulatta | 2 | 105 | 27 | 12 | 20 | 4 |
| TRBV12-2*01\|Mus musculus | 2 | 105 | 27 | 10 | 86 | 3 |
| TRBV12-2*02\|Mus musculus | 2 | 105 | 27 | 10 | 86 | 3 |
| TRBV12-3*01\|Homo sapiens | 2 | 105 | 27 | 12 | 20 | 4 |
| TRBV12-3*01\|Macaca mulatta | 2 | 105 | 27 | 12 | 20 | 4 |
| TRBV12-4*01\|Homo sapiens | 2 | 105 | 27 | 12 | 20 | 4 |
| TRBV12-4*01\|Macaca mulatta | 2 | 105 | 27 | 12 | 20 | 4 |
| TRBV12-4*02\|Homo sapiens | 2 | 105 | 27 | 12 | 20 | 4 |
| TRBV12-5*01\|Homo sapiens | 2 | 105 | 27 | 12 | 20 | 4 |
| TRBV13*01\|Homo sapiens | 2 | 105 | 27 | 10 | 86 | 3 |
| TRBV13*01\|Macaca mulatta | 2 | 105 | 27 | 10 | 86 | 3 |
| TRBV13*02\|Homo sapiens | 2 | 105 | 27 | 10 | 86 | 3 |
| TRBV13-1*01\|Mus musculus | 1 | 105 | 78 | 75 | 86 | 2 |
| TRBV13-1*02\|Mus musculus | 1 | 105 | 78 | 75 | 86 | 2 |
| TRBV13-2*01\|Mus musculus | 1 | 105 | 78 | 75 | 86 | 2 |
| TRBV13-2*02\|Mus musculus | 1 | 105 | 78 | 75 | 86 | 2 |
| TRBV13-2*03\|Mus musculus | 1 | 105 | 78 | 75 | 86 | 2 |
| TRBV13-2*04\|Mus musculus | 1 | 105 | 78 | 75 | 86 | 2 |
| TRBV13-2*05\|Mus musculus | 1 | 105 | 78 | 75 | 86 | 2 |
| TRBV13-3*01\|Mus musculus | 1 | 105 | 78 | 75 | 86 | 2 |
| TRBV14*01\|Homo sapiens | 2 | 105 | 27 | 12 | 20 | 4 |
| TRBV14*01\|Macaca mulatta | 2 | 105 | 27 | 12 | 20 | 4 |
| TRBV14*01\|Mus musculus | 2 | 105 | 27 | 12 | 20 | 4 |
| TRBV14*02\|Homo sapiens | 2 | 105 | 27 | 12 | 20 | 4 |
| TRBV15*01\|Homo sapiens | 2 | 105 | 27 | 75 | 86 | 2 |
| TRBV15*01\|Macaca mulatta | 2 | 105 | 27 | 75 | 86 | 2 |
| TRBV15*01\|Mus musculus | 2 | 105 | 27 | 12 | 20 | 4 |
| TRBV15*02\|Homo sapiens | 2 | 105 | 27 | 75 | 86 | 2 |
| TRBV15*03\|Homo sapiens | 2 | 105 | 27 | 75 | 86 | 2 |
| TRBV16*01\|Canis lupus familiaris | 2 | 105 | 27 | 6 | 20 | 5 |
| TRBV16*01\|Homo sapiens | 2 | 105 | 27 | 6 | 20 | 5 |
| TRBV16*01\|Macaca mulatta | 2 | 105 | 27 | 6 | 20 | 5 |
| TRBV16*01\|Mus musculus | 2 | 105 | 27 | 12 | 20 | 4 |
| TRBV16*02\|Mus musculus | 2 | 105 | 27 | 12 | 20 | 4 |
| TRBV16*03\|Homo sapiens | 2 | 105 | 27 | 6 | 20 | 5 |
| TRBV16*03\|Mus musculus | 2 | 105 | 27 | 12 | 20 | 4 |
| TRBV16*04\|Mus musculus | 2 | 105 | 27 | 12 | 20 | 4 |
| TRBV17*01\|Mus musculus | 2 | 105 | 27 | 75 | 86 | 2 |
| TRBV18*01\|Canis lupus familiaris | 2 | 105 | 27 | 12 | 20 | 4 |
| TRBV18*01\|Homo sapiens | 2 | 105 | 27 | 12 | 20 | 4 |
| TRBV18*01\|Macaca mulatta | 2 | 105 | 27 | 12 | 20 | 4 |
| TRBV19*01\|Homo sapiens | 1 | 105 | 78 | 75 | 86 | 2 |
| TRBV19*01\|Macaca mulatta | 1 | 105 | 78 | 75 | 86 | 2 |
| TRBV19*01\|Mus musculus | 1 | 105 | 78 | 75 | 86 | 2 |
| TRBV19*02\|Homo sapiens | 1 | 105 | 78 | 75 | 86 | 2 |
| TRBV19*02\|Mus musculus | 1 | 105 | 78 | 75 | 86 | 2 |
| TRBV19*03\|Homo sapiens | 1 | 105 | 78 | 75 | 86 | 2 |
| TRBV19*03\|Mus musculus | 1 | 105 | 78 | 75 | 86 | 2 |
| TRBV1S3*01\|Oncorhynchus mykiss | 0 | 0 | 0 | 0 | 0 | 0 |
| TRBV1S5*01\|Oncorhynchus mykiss | 0 | 0 | 0 | 0 | 0 | 0 |
| TRBV2*01\|Homo sapiens | 2 | 105 | 27 | 6 | 20 | 5 |
| TRBV2*01\|Mus musculus | 2 | 105 | 27 | 10 | 86 | 3 |
| TRBV2*02\|Homo sapiens | 2 | 105 | 27 | 6 | 20 | 5 |
| TRBV2*03\|Homo sapiens | 2 | 105 | 27 | 6 | 20 | 5 |
| TRBV2-1*01\|Macaca mulatta | 2 | 105 | 27 | 6 | 20 | 5 |
| TRBV2-2*01\|Macaca mulatta | 2 | 105 | 27 | 6 | 20 | 5 |
| TRBV2-3*01\|Macaca mulatta | 2 | 105 | 27 | 6 | 20 | 5 |
| TRBV20*01\|Canis lupus familiaris | 0 | 0 | 0 | 1 | 3 | 0 |
| TRBV20-1*01\|Homo sapiens | 3 | 9 | 6 | 2 | 3 | 7 |
| TRBV20-1*01\|Macaca mulatta | 3 | 9 | 6 | 2 | 3 | 7 |
| TRBV20-1*02\|Homo sapiens | 3 | 9 | 6 | 2 | 3 | 7 |
| TRBV20-1*03\|Homo sapiens | 3 | 9 | 6 | 2 | 3 | 7 |
| TRBV20-1*04\|Homo sapiens | 3 | 9 | 6 | 2 | 3 | 7 |
| TRBV20-1*05\|Homo sapiens | 3 | 9 | 6 | 2 | 3 | 7 |
| TRBV20-1*06\|Homo sapiens | 3 | 9 | 6 | 2 | 3 | 7 |
| TRBV20-1*07\|Homo sapiens | 3 | 9 | 6 | 2 | 3 | 7 |
| TRBV21-1*01\|Macaca mulatta | 2 | 105 | 27 | 6 | 20 | 5 |
| TRBV22*01\|Canis lupus familiaris | 1 | 105 | 78 | 75 | 86 | 2 |
| TRBV23*01\|Mus musculus | 2 | 105 | 27 | 6 | 20 | 5 |
| TRBV23-1*01\|Macaca mulatta | 2 | 105 | 27 | 6 | 20 | 5 |
| TRBV24*01\|Canis lupus familiaris | 1 | 105 | 78 | 75 | 86 | 2 |
| TRBV24*01\|Mus musculus | 2 | 105 | 27 | 6 | 20 | 5 |
| TRBV24*02\|Mus musculus | 2 | 105 | 27 | 6 | 20 | 5 |
| TRBV24*03\|Mus musculus | 2 | 105 | 27 | 6 | 20 | 5 |
| TRBV24*04\|Mus musculus | 2 | 105 | 27 | 6 | 20 | 5 |
| TRBV24-1*01\|Homo sapiens | 1 | 105 | 78 | 75 | 86 | 2 |
| TRBV24-1*01\|Macaca mulatta | 1 | 105 | 78 | 75 | 86 | 2 |
| TRBV25*01\|Canis lupus familiaris | 1 | 105 | 78 | 75 | 86 | 2 |
| TRBV25-1*01\|Homo sapiens | 1 | 105 | 78 | 75 | 86 | 2 |
| TRBV25-1*01\|Macaca mulatta | 1 | 105 | 78 | 75 | 86 | 2 |
| TRBV26*01\|Mus musculus | 2 | 105 | 27 | 6 | 20 | 5 |
| TRBV26*02\|Mus musculus | 2 | 105 | 27 | 6 | 20 | 5 |
| TRBV27*01\|Homo sapiens | 1 | 105 | 78 | 75 | 86 | 2 |
| TRBV27*01\|Macaca mulatta | 1 | 105 | 78 | 75 | 86 | 2 |
| TRBV28*01\|Homo sapiens | 1 | 105 | 78 | 75 | 86 | 2 |
| TRBV28*01\|Macaca mulatta | 1 | 105 | 78 | 75 | 86 | 2 |
| TRBV29*01\|Mus musculus | 1 | 105 | 78 | 75 | 86 | 2 |
| TRBV29*02\|Mus musculus | 1 | 105 | 78 | 75 | 86 | 2 |
| TRBV29-1*01\|Homo sapiens | 2 | 105 | 27 | 0 | 3 | 0 |
| TRBV29-1*01\|Macaca mulatta | 2 | 105 | 27 | 0 | 3 | 0 |
| TRBV29-1*02\|Homo sapiens | 2 | 105 | 27 | 0 | 3 | 0 |
| TRBV3*01\|Mus musculus | 2 | 105 | 27 | 6 | 20 | 5 |
| TRBV3*02\|Mus musculus | 2 | 105 | 27 | 6 | 20 | 5 |
| TRBV3-1*01\|Canis lupus familiaris | 2 | 105 | 27 | 10 | 86 | 3 |
| TRBV3-1*01\|Homo sapiens | 2 | 105 | 27 | 10 | 86 | 3 |
| TRBV3-1*01\|Macaca mulatta | 1 | 105 | 78 | 10 | 86 | 3 |
| TRBV3-1*02\|Homo sapiens | 2 | 105 | 27 | 10 | 86 | 3 |
| TRBV3-2*01\|Canis lupus familiaris | 2 | 105 | 27 | 10 | 86 | 3 |
| TRBV3-2*01\|Macaca mulatta | 2 | 105 | 27 | 10 | 86 | 3 |
| TRBV3-3*01\|Macaca mulatta | 1 | 105 | 78 | 10 | 86 | 3 |
| TRBV3-4*01\|Macaca mulatta | 2 | 105 | 27 | 10 | 86 | 3 |
| TRBV30*01\|Canis lupus familiaris | 0 | 0 | 0 | 4 | 4 | 1 |
| TRBV30*01\|Homo sapiens | 4 | 9 | 3 | 4 | 4 | 1 |
| TRBV30*01\|Macaca mulatta | 4 | 9 | 3 | 4 | 4 | 1 |
| TRBV30*01\|Mus musculus | 0 | 0 | 0 | 0 | 3 | 0 |
| TRBV30*02\|Homo sapiens | 4 | 9 | 3 | 4 | 4 | 1 |
| TRBV30*04\|Homo sapiens | 4 | 9 | 3 | 4 | 4 | 1 |
| TRBV30*05\|Homo sapiens | 4 | 9 | 3 | 4 | 4 | 1 |
| TRBV31*01\|Mus musculus | 0 | 0 | 0 | 4 | 4 | 1 |
| TRBV31*02\|Mus musculus | 0 | 0 | 0 | 4 | 4 | 1 |
| TRBV4*01\|Mus musculus | 1 | 105 | 78 | 10 | 86 | 3 |
| TRBV4*02\|Mus musculus | 1 | 105 | 78 | 10 | 86 | 3 |
| TRBV4-1*01\|Canis lupus familiaris | 2 | 105 | 27 | 10 | 86 | 3 |
| TRBV4-1*01\|Homo sapiens | 1 | 105 | 78 | 10 | 86 | 3 |
| TRBV4-1*01\|Macaca mulatta | 1 | 105 | 78 | 10 | 86 | 3 |
| TRBV4-1*02\|Homo sapiens | 1 | 105 | 78 | 10 | 86 | 3 |
| TRBV4-2*01\|Canis lupus familiaris | 2 | 105 | 27 | 10 | 86 | 3 |
| TRBV4-2*01\|Homo sapiens | 1 | 105 | 78 | 10 | 86 | 3 |
| TRBV4-2*01\|Macaca mulatta | 1 | 105 | 78 | 10 | 86 | 3 |
| TRBV4-2*02\|Homo sapiens | 1 | 105 | 78 | 10 | 86 | 3 |
| TRBV4-3*01\|Canis lupus familiaris | 2 | 105 | 27 | 10 | 86 | 3 |
| TRBV4-3*01\|Homo sapiens | 1 | 105 | 78 | 10 | 86 | 3 |
| TRBV4-3*01\|Macaca mulatta | 1 | 105 | 78 | 10 | 86 | 3 |
| TRBV4-3*02\|Homo sapiens | 1 | 105 | 78 | 10 | 86 | 3 |
| TRBV4-3*03\|Homo sapiens | 1 | 105 | 78 | 10 | 86 | 3 |
| TRBV4-3*04\|Homo sapiens | 1 | 105 | 78 | 10 | 86 | 3 |
| TRBV5*01\|Mus musculus | 1 | 105 | 78 | 10 | 86 | 3 |
| TRBV5*02\|Mus musculus | 1 | 105 | 78 | 10 | 86 | 3 |
| TRBV5*03\|Mus musculus | 1 | 105 | 78 | 10 | 86 | 3 |
| TRBV5*04\|Mus musculus | 1 | 105 | 78 | 10 | 86 | 3 |
| TRBV5*05\|Mus musculus | 1 | 105 | 78 | 10 | 86 | 3 |
| TRBV5-1*01\|Homo sapiens | 2 | 105 | 27 | 10 | 86 | 3 |
| TRBV5-1*02\|Homo sapiens | 2 | 105 | 27 | 10 | 86 | 3 |
| TRBV5-10*01\|Macaca mulatta | 2 | 105 | 27 | 10 | 86 | 3 |
| TRBV5-2*01\|Canis lupus familiaris | 2 | 105 | 27 | 10 | 86 | 3 |
| TRBV5-4*01\|Canis lupus familiaris | 2 | 105 | 27 | 10 | 86 | 3 |
| TRBV5-4*01\|Homo sapiens | 2 | 105 | 27 | 10 | 86 | 3 |
| TRBV5-4*01\|Macaca mulatta | 2 | 105 | 27 | 10 | 86 | 3 |
| TRBV5-4*02\|Homo sapiens | 2 | 105 | 27 | 10 | 86 | 3 |
| TRBV5-4*03\|Homo sapiens | 2 | 105 | 27 | 10 | 86 | 3 |
| TRBV5-4*04\|Homo sapiens | 0 | 0 | 0 | 10 | 86 | 3 |
| TRBV5-5*01\|Homo sapiens | 2 | 105 | 27 | 10 | 86 | 3 |
| TRBV5-5*01\|Macaca mulatta | 2 | 105 | 27 | 10 | 86 | 3 |
| TRBV5-5*02\|Homo sapiens | 2 | 105 | 27 | 10 | 86 | 3 |
| TRBV5-5*03\|Homo sapiens | 2 | 105 | 27 | 10 | 86 | 3 |
| TRBV5-6*01\|Homo sapiens | 2 | 105 | 27 | 10 | 86 | 3 |
| TRBV5-6*01\|Macaca mulatta | 2 | 105 | 27 | 10 | 86 | 3 |
| TRBV5-7*01\|Macaca mulatta | 2 | 105 | 27 | 10 | 86 | 3 |
| TRBV5-8*01\|Homo sapiens | 2 | 105 | 27 | 10 | 86 | 3 |
| TRBV5-8*01\|Macaca mulatta | 2 | 105 | 27 | 10 | 86 | 3 |
| TRBV5-8*02\|Homo sapiens | 2 | 105 | 27 | 10 | 86 | 3 |
| TRBV5-9*01\|Macaca mulatta | 2 | 105 | 27 | 10 | 86 | 3 |
| TRBV6-1*01\|Homo sapiens | 1 | 105 | 78 | 75 | 86 | 2 |
| TRBV6-1*01\|Macaca mulatta | 1 | 105 | 78 | 75 | 86 | 2 |
| TRBV6-2*01\|Homo sapiens | 1 | 105 | 78 | 75 | 86 | 2 |
| TRBV6-2*01\|Macaca mulatta | 1 | 105 | 78 | 75 | 86 | 2 |
| TRBV6-3*01\|Homo sapiens | 1 | 105 | 78 | 75 | 86 | 2 |
| TRBV6-3*01\|Macaca mulatta | 1 | 105 | 78 | 75 | 86 | 2 |
| TRBV6-4*01\|Homo sapiens | 1 | 105 | 78 | 75 | 86 | 2 |
| TRBV6-4*01\|Macaca mulatta | 1 | 105 | 78 | 75 | 86 | 2 |
| TRBV6-4*02\|Homo sapiens | 1 | 105 | 78 | 75 | 86 | 2 |
| TRBV6-5*01\|Homo sapiens | 1 | 105 | 78 | 75 | 86 | 2 |
| TRBV6-5*01\|Macaca mulatta | 1 | 105 | 78 | 75 | 86 | 2 |
| TRBV6-6*01\|Homo sapiens | 1 | 105 | 78 | 75 | 86 | 2 |
| TRBV6-6*01\|Macaca mulatta | 1 | 105 | 78 | 75 | 86 | 2 |
| TRBV6-6*02\|Homo sapiens | 1 | 105 | 78 | 75 | 86 | 2 |
| TRBV6-6*03\|Homo sapiens | 1 | 105 | 78 | 75 | 86 | 2 |
| TRBV6-6*04\|Homo sapiens | 1 | 105 | 78 | 75 | 86 | 2 |
| TRBV6-6*05\|Homo sapiens | 1 | 105 | 78 | 75 | 86 | 2 |
| TRBV6-7*01\|Macaca mulatta | 1 | 105 | 78 | 75 | 86 | 2 |
| TRBV6-8*01\|Homo sapiens | 1 | 105 | 78 | 0 | 4 | 2 |
| TRBV6-8*01\|Macaca mulatta | 1 | 105 | 78 | 75 | 86 | 2 |
| TRBV6-9*01\|Homo sapiens | 1 | 105 | 78 | 75 | 86 | 2 |
| TRBV7*01\|Canis lupus familiaris | 2 | 105 | 27 | 12 | 20 | 4 |
| TRBV7-10*01\|Macaca mulatta | 2 | 105 | 27 | 6 | 20 | 5 |
| TRBV7-2*01\|Homo sapiens | 2 | 105 | 27 | 12 | 20 | 4 |
| TRBV7-2*01\|Macaca mulatta | 0 | 0 | 0 | 12 | 20 | 4 |
| TRBV7-2*02\|Homo sapiens | 2 | 105 | 27 | 12 | 20 | 4 |
| TRBV7-2*03\|Homo sapiens | 2 | 105 | 27 | 12 | 20 | 4 |
| TRBV7-2*04\|Homo sapiens | 2 | 105 | 27 | 12 | 20 | 4 |
| TRBV7-3*01\|Homo sapiens | 2 | 105 | 27 | 12 | 20 | 4 |
| TRBV7-3*01\|Macaca mulatta | 2 | 105 | 27 | 12 | 20 | 4 |
| TRBV7-3*02\|Homo sapiens | 2 | 105 | 27 | 12 | 20 | 4 |
| TRBV7-3*03\|Homo sapiens | 2 | 105 | 27 | 12 | 20 | 4 |
| TRBV7-3*04\|Homo sapiens | 2 | 105 | 27 | 12 | 20 | 4 |
| TRBV7-3*05\|Homo sapiens | 2 | 105 | 27 | 12 | 20 | 4 |
| TRBV7-4*01\|Homo sapiens | 2 | 105 | 27 | 12 | 20 | 4 |
| TRBV7-4*01\|Macaca mulatta | 2 | 105 | 27 | 12 | 20 | 4 |
| TRBV7-5*01\|Macaca mulatta | 2 | 105 | 27 | 12 | 20 | 4 |
| TRBV7-6*01\|Homo sapiens | 2 | 105 | 27 | 12 | 20 | 4 |
| TRBV7-6*01\|Macaca mulatta | 2 | 105 | 27 | 12 | 20 | 4 |
| TRBV7-6*02\|Homo sapiens | 2 | 105 | 27 | 12 | 20 | 4 |
| TRBV7-7*01\|Homo sapiens | 2 | 105 | 27 | 12 | 20 | 4 |
| TRBV7-7*01\|Macaca mulatta | 2 | 105 | 27 | 12 | 20 | 4 |
| TRBV7-7*02\|Homo sapiens | 2 | 105 | 27 | 12 | 20 | 4 |
| TRBV7-8*01\|Homo sapiens | 2 | 105 | 27 | 12 | 20 | 4 |
| TRBV7-8*02\|Homo sapiens | 2 | 105 | 27 | 12 | 20 | 4 |
| TRBV7-8*03\|Homo sapiens | 2 | 105 | 27 | 12 | 20 | 4 |
| TRBV7-9*01\|Homo sapiens | 2 | 105 | 27 | 6 | 20 | 5 |
| TRBV7-9*01\|Macaca mulatta | 2 | 105 | 27 | 12 | 20 | 4 |
| TRBV7-9*02\|Homo sapiens | 2 | 105 | 27 | 6 | 20 | 5 |
| TRBV7-9*03\|Homo sapiens | 2 | 105 | 27 | 6 | 20 | 5 |
| TRBV7-9*04\|Homo sapiens | 2 | 105 | 27 | 6 | 20 | 5 |
| TRBV7-9*05\|Homo sapiens | 2 | 105 | 27 | 6 | 20 | 5 |
| TRBV7-9*06\|Homo sapiens | 2 | 105 | 27 | 6 | 20 | 5 |
| TRBV7-9*07\|Homo sapiens | 0 | 0 | 0 | 6 | 20 | 5 |
| TRBV9*01\|Homo sapiens | 2 | 105 | 27 | 10 | 86 | 3 |
| TRBV9*01\|Macaca mulatta | 2 | 105 | 27 | 10 | 86 | 3 |
| TRBV9*02\|Homo sapiens | 2 | 105 | 27 | 10 | 86 | 3 |
| TRBV9*03\|Homo sapiens | 2 | 105 | 27 | 10 | 86 | 3 |

**Table S5:** Alignment errors. All the TCR α and β functional variable gene present in the IMGT/GENE-DB database for which our HMM-based alignment routine could not produce a proper alignment are reported.

| **Alignment errors** | |
| --- | --- |
| **TRA genes** | **TRB genes** |
| TRAV6-6*03\|Mus musculus | TRBV29-1*03\|Homo sapiens |
| TRAV13D-3*02\|Mus musculus | TRBV9S1*01\|Oncorhynchus mykiss |
| TRAV13N-3*01\|Mus musculus | TRBV2S8*01\|Oncorhynchus mykiss |
| TRAV7D-2*03\|Mus musculus | TRBV8S1*01\|Oncorhynchus mykiss |
| TRBV2S19*01\|Oncorhynchus mykiss | TRBV2S7*01\|Oncorhynchus mykiss |
| TRBV2S20*01\|Oncorhynchus mykiss | TRBV3S1*01\|Oncorhynchus mykiss |
| TRBV2S3*01\|Oncorhynchus mykiss | TRBV1S2*01\|Oncorhynchus mykiss |
| TRBV7S1*01\|Oncorhynchus mykiss | TRBV2S17*01\|Oncorhynchus mykiss |
| TRBV7S2*01\|Oncorhynchus mykiss | TRBV1S1*01\|Oncorhynchus mykiss |
| TRBV7S3*01\|Oncorhynchus mykiss | TRBV20*01\|Mus musculus |
| TRBV7S4*01\|Oncorhynchus mykiss | TRBV2S9*01\|Oncorhynchus mykiss |
|  | TRBV2S14*01\|Oncorhynchus mykiss |
|  | TRBV2S6*01\|Oncorhynchus mykiss |
|  | TRBV8S3*01\|Oncorhynchus mykiss |
|  | TRBV2S13*01\|Oncorhynchus mykiss |
|  | TRBV20*02\|Mus musculus |
|  | TRBV2S5*01\|Oncorhynchus mykiss |
|  | TRBV3S2*01\|Oncorhynchus mykiss |
|  | TRBV2S23*01\|Oncorhynchus mykiss |
|  | TRBV1S4*01\|Oncorhynchus mykiss |
|  | TRBV2S18*01\|Oncorhynchus mykiss |
|  | TRBV2S4*01\|Oncorhynchus mykiss |
|  | TRBV4S1*01\|Oncorhynchus mykiss |
|  | TRBV2S27*01\|Oncorhynchus mykiss |
|  | TRBV2S10*01\|Oncorhynchus mykiss |
|  | TRBV29*01\|Canis lupus familiaris |
|  | TRBV2S24*01\|Oncorhynchus mykiss |
|  | TRBV2S1*01\|Oncorhynchus mykiss |
|  | TRBV16*02\|Homo sapiens |
|  | TRBV6S1*01\|Oncorhynchus mykiss |
|  | TRBV5S1*01\|Oncorhynchus mykiss |
|  | TRBV10S1*01\|Oncorhynchus mykiss |
|  | TRBV2S12*01\|Oncorhynchus mykiss |
|  | TRBV2S16*01\|Oncorhynchus mykiss |
|  | TRBV8S2*01\|Oncorhynchus mykiss |

**Table S6:** Accuracy of LYRA models using a leave-one-out procedure with 95% identity threshold. The region-specific accuracy of both raw and refined models generated by LYRA using a leave-one-out procedure and discarding templates with more than 95% identity with the target.

| RMSD  Region | RMSD (raw) | STD (raw) | RMSD (refined) | STD (refined) |
| --- | --- | --- | --- | --- |
| Total | 1.48 | 0.39 | 1.54 | 0.35 |
| FW | 1.03 | 0.35 | 1.15 | 0.31 |
| ABS | 2.13 | 0.67 | 2.14 | 0.61 |
| Total A | 1.27 | 0.48 | 1.34 | 0.41 |
| FW A | 0.74 | 0.37 | 0.91 | 0.32 |
| A loops | 2.11 | 0.87 | 2.11 | 0.73 |
| A1 | 1.29 | 0.72 | 1.47 | 0.64 |
| A2 | 1.37 | 1.18 | 1.50 | 0.95 |
| A3 | 2.61 | 1.24 | 2.57 | 1.05 |
| Total B | 1.21 | 0.52 | 1.31 | 0.45 |
| FW B | 0.69 | 0.33 | 0.88 | 0.27 |
| B loops | 1.73 | 0.89 | 1.78 | 0.80 |
| B1 | 0.81 | 0.54 | 1.08 | 0.43 |
| B2 | 1.16 | 1.00 | 1.29 | 0.90 |
| B3 | 2.54 | 1.29 | 2.51 | 1.17 |

FW: Framework, ABS: Antigen binding site**.**

**Table S7:** Accuracy of LYRA models using a leave-one-out procedure with 90% identity threshold. The region-specific accuracy of both raw and refined models generated by LYRA using a leave-one-out procedure and discarding templates with more than 90% identity with the target.

| RMSD  Region | RMSD (raw) | STD (raw) | RMSD (refined) | STD (refined) |
| --- | --- | --- | --- | --- |
| Total | 1.54 | 0.37 | 1.58 | 0.34 |
| FW | 1.08 | 0.32 | 1.19 | 0.30 |
| ABS | 2.21 | 0.60 | 2.20 | 0.57 |
| Total A | 1.33 | 0.49 | 1.38 | 0.44 |
| FW A | 0.76 | 0.37 | 0.92 | 0.33 |
| A loops | 2.22 | 0.88 | 2.19 | 0.77 |
| A1 | 1.29 | 0.71 | 1.49 | 0.63 |
| A2 | 1.40 | 1.19 | 1.55 | 0.99 |
| A3 | 2.80 | 1.26 | 2.68 | 1.11 |
| Total B | 1.27 | 0.53 | 1.34 | 0.47 |
| FW B | 0.73 | 0.33 | 0.89 | 0.28 |
| B loops | 1.84 | 0.86 | 1.86 | 0.79 |
| B1 | 0.80 | 0.53 | 1.11 | 0.45 |
| B2 | 1.18 | 1.00 | 1.32 | 0.90 |
| B3 | 2.77 | 1.24 | 2.66 | 1.14 |

FW: Framework, ABS: Antigen binding site

**Table S8:** Accuracy of LYRA on the independent TCR dataset. The region-specific accuracy of the refined models generated by LYRA on a set of 4 novel TCRs not used for developing the method is reported. The 4X6B example is described with more details in the main text of this work. The “Mean ± STD” and “Mean ± STD

LOO 90%” refer to the RMSD of the corresponding region in the independent dataset and in the 90% threshold leave-one-out refined models from table S7, respectively.

| PDB  Region | 4ONH | 4QOK | 4QRP | 4X6B | Mean±STD | Mean±STD  LOO 90% |
| --- | --- | --- | --- | --- | --- | --- |
| Total | 1.80 | 1.49 | 1.66 | 1.61 | 1.64 ±0.13 | 1.58±0.34 |
| FW | 1.66 | 0.63 | 1.15 | 0.58 | 1.01 ±0.51 | 1.19±0.30 |
| ABS | 2.06 | 2.56 | 2.45 | 2.75 | 2.46 ±0.29 | 2.20±0.57 |
| Total A | 1.64 | 0.55 | 1.67 | 1.67 | 1.38 ±0.56 | 1.38±0.44 |
| FW A | 1.29 | 0.41 | 0.93 | 0.43 | 0.77 ±0.42 | 0.92±0.33 |
| A loops | 2.34 | 0.85 | 2.95 | 3.17 | 2.33 ±1.05 | 2.19±0.77 |
| A1 | 2.30 | 0.56 | 1.64 | 0.65 | 1.29 ±0.83 | 1.49±0.63 |
| A2 | 2.20 | 0.47 | 4.53 | 1.64 | 2.21 ±1.71 | 1.55±0.99 |
| A3 | 2.43 | 1.18 | 2.47 | 4.40 | 2.62 ±1.33 | 2.68±1.11 |
| Total B | 1.44 | 1.96 | 1.09 | 1.59 | 1.52 ±0.36 | 1.34±0.47 |
| FW B | 1.04 | 0.62 | 0.49 | 0.53 | 0.67 ±0.25 | 0.89±0.28 |
| B loops | 1.98 | 3.16 | 1.69 | 2.56 | 2.35 ±0.65 | 1.86±0.79 |
| B1 | 1.02 | 2.31 | 1.11 | 0.56 | 1.25 ±0.75 | 1.11±0.45 |
| B2 | 0.65 | 2.87 | 1.47 | 0.70 | 1.42 ±1.04 | 1.32±0.90 |
| B3 | 3.36 | 3.99 | 2.24 | 4.28 | 3.47 ±0.90 | 2.66±1.14 |

FW: Framework, ABS: Antigen binding site
